# From Pollutant Removal to Renewable Energy: MoS_2_-Enhanced P25-Graphene Photocatalysts for Malathion Degradation and H_2_ Evolution

**DOI:** 10.3390/ma18112602

**Published:** 2025-06-03

**Authors:** Cristian Martínez-Perales, Abniel Machín, Pedro J. Berríos-Rolón, Paola Sampayo, Enrique Nieves, Loraine Soto-Vázquez, Edgard Resto, Carmen Morant, José Ducongé, María C. Cotto, Francisco Márquez

**Affiliations:** 1 Nanomaterials Research Group, Department of Natural Sciences and Technology, Division of Natural Sciences and Technology, Universidad Ana G. Méndez-Gurabo Campus, Gurabo, PR 00778, USA; martinezc4@uagm.edu (C.M.-P.); berriosp1@uagm.edu (P.J.B.-R.); jduconge@uagm.edu (J.D.); mcotto48@uagm.edu (M.C.C.); 2 Environmental Catalysis Research Lab, Division of Science, Technology and Environment, Cupey Campus, Universidad Ana G. Méndez, Cupey, PR 00926, USA; 3 Department of Pharmaceutical Sciences, Nova Southeastern University, Puerto Rico Campus, San Juan, PR 00926, USA; nenrique@nova.edu; 4 Materials Characterization Center Inc., Molecular Sciences Research Center, University of Puerto Rico, San Juan, PR 00926, USA; loraine.soto@mcc.com.pr (L.S.-V.); edgar.resto@upr.edu (E.R.); 5 Instituto de Ciencia de Materiales Nicolás Cabrera, Department of Applied Physics, Autonomous University of Madrid, 28049 Madrid, Spain; c.morant@uam.es

**Keywords:** photodegradation, photocatalytic hydrogen evolution, malathion, rGO, MoS_2_ nanocomposite

## Abstract

The widespread presence of pesticides—especially malathion—in aquatic environments presents a major obstacle to conventional remediation strategies, while the ongoing global energy crisis underscores the urgency of developing renewable energy sources such as hydrogen. In this context, photocatalytic water splitting emerges as a promising approach, though its practical application remains limited by poor charge carrier dynamics and insufficient visible-light utilization. Herein, we report the design and evaluation of a series of TiO_2_-based ternary nanocomposites comprising commercial P25 TiO_2_, reduced graphene oxide (rGO), and molybdenum disulfide (MoS_2_), with MoS_2_ loadings ranging from 1% to 10% by weight. The photocatalysts were fabricated via a two-step method: hydrothermal integration of rGO into P25 followed by solution-phase self-assembly of exfoliated MoS_2_ nanosheets. The composites were systematically characterized using X-ray diffraction (XRD), Raman spectroscopy, transmission electron microscopy (TEM), UV-Vis diffuse reflectance spectroscopy (DRS), and photoluminescence (PL) spectroscopy. Photocatalytic activity was assessed through two key applications: the degradation of malathion (20 mg/L) under simulated solar irradiation and hydrogen evolution from water in the presence of sacrificial agents. Quantification was performed using UV-Vis spectroscopy, gas chromatography–mass spectrometry (GC-MS), and thermal conductivity detection (GC-TCD). Results showed that the integration of rGO significantly enhanced surface area and charge mobility, while MoS_2_ served as an effective co-catalyst, promoting interfacial charge separation and acting as an active site for hydrogen evolution. Nearly complete malathion degradation (~100%) was achieved within two hours, and hydrogen production reached up to 6000 µmol g^−1^ h^−1^ under optimal MoS_2_ loading. Notably, photocatalytic performance declined with higher MoS_2_ content due to recombination effects. Overall, this work demonstrates the synergistic enhancement provided by rGO and MoS_2_ in a stable P25-based system and underscores the viability of such ternary nanocomposites for addressing both environmental remediation and sustainable energy conversion challenges.

## 1. Introduction

Environmental contamination caused by pesticides has become a growing concern due to their widespread use in agriculture and their persistence in water bodies [1]. Among these contaminants, malathion, an organophosphate pesticide, is particularly problematic due to its toxicity, bioaccumulation potential, and resistance to natural degradation [2,3]. Traditional water treatment methods, such as adsorption, coagulation, and biological degradation, often fail to remove malathion efficiently, requiring the development of alternative approaches [4]. At the same time, the global energy crisis and the urgent need for sustainable fuel sources have driven increasing interest in hydrogen production as an environmentally friendly alternative to fossil fuels [5,6,7]. Photocatalytic water splitting has emerged as a promising method for producing hydrogen in a clean and renewable manner [8,9]. However, challenges related to inefficient charge separation, limited light absorption, and low reaction rates had hindered its large-scale application [10].

Despite the emergence of more advanced visible-light-active semiconductors, P25 TiO_2_ remains one of the most widely used benchmark photocatalysts due to its commercial availability, well-defined anatase/rutile composition, chemical stability, and reproducibility. These features make it a valuable reference platform for assessing the impact of co-catalyst and carbon-based modifications, particularly in systems aimed at simultaneous environmental and energy applications. To address these issues, the present study explored the use of TiO_2_-based nanocomposites, specifically P25-graphene nanocatalysts modified with different MoS_2_ loadings (1%, 3%, 5%, and 10%), for two key applications: the photocatalytic degradation of malathion under simulated solar irradiation and the photocatalytic production of hydrogen from water. The incorporation of graphene into P25 TiO_2_ has been previously shown to improve charge carrier separation, enhance surface area, and facilitate electron transport, leading to increased photocatalytic efficiency [11,12,13]. Additionally, MoS_2_, a transition metal dichalcogenide with a narrow bandgap (~1.8 eV) and high electron mobility, has demonstrated potential as a co-catalyst in photocatalysis by promoting charge transfer and providing active sites for hydrogen evolution reactions [14,15]. However, no previous study has systematically evaluated the effect of MoS_2_ loading in P25-graphene composites for the dual purpose of pollutant degradation and hydrogen generation.

In this study, a series of P25-graphene-MoS_2_ nanocomposites were synthesized and characterized to investigate their structural, morphological, and optical properties. X-ray diffraction (XRD) was used to confirm phase composition, while Raman spectroscopy provided insight into the structural interactions among P25, graphene, and MoS_2_. Transmission electron microscopy (TEM) allowed for the characterization of dispersion and morphology, and UV-Vis reflectance spectroscopy (DRS) was employed to assess optical absorption properties. Photoluminescence (PL) spectroscopy was further used to evaluate charge carrier recombination behavior. The photocatalytic degradation of malathion was performed under simulated solar irradiation, and the degradation efficiency was monitored using gas chromatography–mass spectrometry (GC-MS). Additionally, photocatalytic hydrogen production experiments were conducted in an aqueous suspension with sacrificial reagents, and hydrogen evolution rates were quantified using gas chromatography (GC-TCD).

The results demonstrated that the incorporation of graphene and MoS_2_ into P25 TiO_2_ significantly enhanced photocatalytic activity for both malathion degradation and hydrogen evolution. As expected [16], the presence of graphene facilitated charge separation and improved electrical conductivity, reducing electron–hole recombination. MoS_2_ further contributed to the process by acting as an electron acceptor and providing catalytic sites for hydrogen generation. An optimal MoS_2_ loading was identified, beyond which excessive MoS_2_ led to charge recombination and a decline in performance. This work provides new insight into how the compositional tuning of MoS_2_ affects dual-function photocatalysis, revealing a compositional threshold that balances charge transfer and catalytic activity. These findings highlight the importance of nanocomposite composition in optimizing photocatalytic efficiency and provide valuable insights into the design of multifunctional photocatalysts for environmental remediation and sustainable energy production. By clearly identifying the optimal configuration and explaining the trade-offs associated with excessive MoS_2_, this study not only demonstrates the dual functionality of P25-graphene-MoS_2_ nanocomposites but also provides a comprehensive understanding of the synergistic effects among the individual components. By evaluating the impact of MoS_2_ loading, this work contributes to the development of cost-effective and scalable photocatalysts with potential applications in water treatment and renewable hydrogen production. The findings underscore the potential of engineered nanomaterials in addressing critical environmental and energy challenges, paving the way for further advancements in photocatalysis-based technologies.

Beyond demonstrating the feasibility of MoS_2_@P25-rGO nanocomposites for dual-function photocatalysis, this work introduces several novel elements to the field. First, it provides the feasibility of MoS_2_@P25-rGO nanocomposites for simultaneous pollutant degradation and hydrogen production while also contributing several novel elements to the field. Second, it provides a systematic evaluation of MoS_2_ loading and its dual effect on surface area and photocatalytic behavior, identifying a compositional threshold beyond which performance deteriorates. Third, the study establishes mechanistic correlations between structural and spectroscopic characterization (BET, PL, and photocurrent) and functional performance, supporting a charge transfer model involving Z-scheme or type II heterojunctions. Finally, the incorporation of hydroxyl radical formation and scavenger-based validation helps elucidate a more complete reaction mechanism. These innovations offer valuable insight into the design of multifunctional photocatalysts, reinforcing the significance of this ternary system as a scalable and cost-effective platform for environmental and energy applications.

## 2. Materials and Methods

### 2.1. Materials

All chemicals were used as received without further purification, and solutions were prepared using deionized water (Milli-Q water, 18.2 MΩcm^−1^ at 25 °C). The TiO_2_ used in this study was Aeroxide(R) P25 (99.5% purity), provided by Thermo Scientific Chemicals. Reduced graphene oxide (rGO, purified powder), MoS_2_ (powder, <2mm, 98%), N-Methyl-2-pyrrolidone (ReagentPlus^®^, 99%), and malathion (TraceCERT^®^ standard, 99.5%) were provided by Sigma Aldrich (Milwaukee, WI, USA). Sodium sulfite (Na_2_SO_3_) anhydrous for analysis EMSURE^®^ Reag. Ph Eur and sodium sulfide nonahydrate (≥99.99% trace metals basis), used as sacrificial reagents, were provided by Sigma Aldrich (Milwaukee, WI, USA). Hydrogen peroxide (H_2_O_2_, 30% *w*/*w*), methanol (CH_3_OH, HPLC grade, >99.9%), and 0.45 μm syringe filters were also obtained from Sigma Aldrich (Milwaukee, WI, USA). Sodium hydroxide (NaOH, pellets, ACS Certified) and hydrochloric acid (HCl, ACS, 36.5–38%) were provided by Fisher Chemical (Waltham, MA, USA).

### 2.2. Preparation of the TiO_2_-rGO Composite

The synthesis of the TiO_2_-rGO composite was conducted via a hydrothermal method, modifying the approach reported by Perera et al. [17]. To ensure a well-defined composition, the weight ratio (*w*/*w*) of rGO to TiO_2_ (P25) was set at 3%. In a single-step sonication process, rGO was dispersed in 30 mL of deionized water containing 10.5 g of NaOH. This solution was subjected to ultrasonication at 40 kHz for 1 h to achieve a uniform dispersion of rGO. Simultaneously, the TiO_2_ powder was incorporated into the mixture, ensuring homogeneous suspension. After sonication, the dispersion was subjected to magnetic stirring at 500 rpm for 1 h. Subsequently, the resulting TiO_2_-rGO suspension was transferred into a 100 mL Teflon-lined stainless-steel autoclave and subjected to hydrothermal treatment at 120 °C for 24 h under static conditions. This process facilitated the incorporation of rGO onto the TiO_2_ surface while preserving the structural integrity of both materials. Upon completion of the hydrothermal reaction, the obtained gray gel was carefully collected and washed with a 0.1 mol/L HCl solution (prepared using concentrated HCl, 35%, Fisher Scientific). The sample was continuously stirred overnight at room temperature (~25 °C) to ensure thorough removal of NaOH. To purify the material, sequential washing steps were performed. The product was washed five times with deionized water, followed by a final rinse with ethanol to enhance dispersion and minimize agglomeration. Centrifugation (6000 rpm, 10 min) was employed between each washing step to facilitate solid–liquid separation and ensure the removal of unreacted precursors and residual ionic species. Following purification, the material was dried in a vacuum oven at 80 °C for 12 h. Finally, to enhance crystallinity and optimize the physicochemical properties of the composite, the TiO_2_-rGO material was annealed at 300 °C for 60 min in an air atmosphere. This thermal treatment was aimed at improving the interaction between TiO_2_ and rGO while maintaining the integrity of the composite structure.

### 2.3. Preparation of MoS_2_@TiO_2_-rGO Catalysts

The incorporation of exfoliated MoS_2_ into the previously synthesized TiO_2_-rGO composite was carried out via a solution-based self-assembly method. Exfoliated MoS_2_ nanosheets were obtained via liquid-phase exfoliation (LPE) in anhydrous N-methyl-2-pyrrolidone (NMP) [18,19]. The exfoliation process involved ultrasonication at 40 kHz for 6 h in an ice-cooled bath. After sonication, the dispersion was centrifuged at 3000 rpm for 30 min, and the supernatant, containing well-dispersed MoS_2_ nanosheets, was collected and filtered to remove unexfoliated bulk material. The resulting MoS_2_ suspension was washed four times with ethanol and then redispersed in deionized water (pH 7) at a concentration of 1 mg/mL under continuous stirring for 1 h before use. To incorporate exfoliated MoS_2_ into the TiO_2_-rGO matrix, 0.5 g of TiO_2_-rGO was dispersed in 50 mL of deionized water under magnetic stirring at 600 rpm for 1 h. Subsequently, an MoS_2_ suspension (1 mg/mL) was added dropwise in the appropriate amount to obtain composites containing 1%, 3%, 5%, and 10% MoS_2_ by weight relative to TiO_2_-rGO, while maintaining continuous stirring. The resulting mixture was then subjected to ultrasonication at 40 kHz for 30 min to ensure homogeneous distribution and to enhance the interaction between the MoS_2_ nanosheets and the TiO_2_-rGO hybrid structure. Following sonication, the dispersion was stirred for 4 h at room temperature to enhance adhesion and allow van der Waals interactions between the MoS_2_ and rGO layers. After stirring, the MoS_2_-TiO_2_-rGO composite was collected via centrifugation at 8000 rpm for 10 min, washed three times with ethanol/water (1:1 *v*/*v*), and dried in a vacuum oven at 80 °C for 12 h. To improve the structural integrity and enhance the electronic coupling between the components, the dried composite was subjected to a mild thermal annealing step at 250 °C for 2 h under an inert nitrogen atmosphere. This step facilitated the reduction of residual oxygen functional groups in rGO and improved the contact between the MoS_2_ and the TiO_2_-rGO surface without inducing phase transitions.

### 2.4. Characterization Techniques

A comprehensive set of characterization techniques was employed to evaluate the structural, morphological, optical, and chemical properties of the synthesized catalysts. The specific surface area and porosity of the materials were determined using Brunauer–Emmett–Teller (BET) analysis on a Micromeritics ASAP 2020 instrument (Micromeritics Instrument Corporation, Norcross, GA, USA), measuring nitrogen adsorption–desorption isotherms at 77 K. The morphological features and microstructural details of the composites were analyzed by field emission scanning electron microscopy (FESEM) using a JEOL IT-500HR instrument (JEOL, Peabody, MA, USA). Additionally, high-resolution transmission electron microscopy (HRTEM) was conducted on a JEOL JEM 3000F microscope (JEOL, Peabody, MA, USA) operating at 300 kV. The crystalline phases of the synthesized catalysts were characterized by X-ray diffraction (XRD) on a Bruker D8 Advance diffractometer (Bruker, Billerica, MA, USA), operating at 40 kV and 40 mA. Raman spectroscopy was performed using a DXR Thermo Raman Microscope (Thermo Fisher Scientific, Waltham, MA, USA) employing a 532 nm laser source with a power setting of 5 mW and a resolution of 5 cm^−1^. The chemical states were analyzed using X-ray photoelectron spectroscopy (XPS) on an ESCALAB 220i-XL spectrometer (VG Scientific Ltd., Loughborough, UK) employing non-monochromatic Mg Kα radiation of a twin anode at 20 mA and 12 kV. The bandgap estimation was obtained by UV-Vis diffuse reflectance spectroscopy (UV-Vis DRS) on a PerkinElmer Lambda 850 UV-Vis spectrophotometer (PerkinElmer lambda, Waltham, MA, USA). Photoluminescence (PL) spectroscopy was conducted using an Edinburgh FS900 fluorescence spectrometer (Edinburgh Instr., Livingston, Scotland) to evaluate charge carrier recombination and photogenerated electron–hole interactions. The photocatalytic degradation of malathion was characterized by UV-Vis spectroscopy on a PerkinElmer Lambda 850 UV-Vis spectrophotometer. To characterize the degradation intermediates, gas chromatography–mass spectrometry (GC-MS) analysis was performed using a Shimadzu GC 2010 Plus-QP2020 system (Shimadzu Corporation, Kyoto, Japan). The separation of organic compounds was achieved using a 30 m × 0.25 mm i.d. capillary column (Rtx-5MS, Restek Corporation, Bellefonte, PA, USA) with helium (99.999% purity) as the carrier gas.

### 2.5. Photocatalytic Degradation Experiments

Photodegradation experiments were conducted using a 20 ppm solution of malathion mixed with 1.1 g/L of the selected catalyst. The pH of the resulting solution was adjusted to 7 using either sodium hydroxide (NaOH) or hydrochloric acid (HCl). To ensure adsorption/desorption equilibrium between the catalyst and the contaminant, the suspension was stirred in the dark for 30 min. Following this equilibration period, a 1 mL aliquot of a 0.01% hydrogen peroxide (H_2_O_2_) solution was added, and the system was continuously aerated to maintain sufficient oxygen levels. All photocatalytic degradation experiments were performed using a dual 100 W white-light halogen lamp system, which provided a total irradiance of approximately 6300 lux (equivalent to ~75 mW·cm^−2^ at the sample level). This setup simulates the solar spectrum in the 300–800 nm range under standard AM 1.5 terrestrial conditions [20]. Upon initiating irradiation, the experiment proceeded for 120 min at a controlled temperature of 22 °C, with 5 mL samples collected at 10 min intervals. Each collected sample was passed through a 0.45 μm membrane filter to remove any catalyst residues and subsequently analyzed using UV-visible spectroscopy. All photocatalytic degradation experiments were performed in triplicate under identical conditions, and the results were averaged for each time point. The relative standard deviation across replicates was consistently below 4%, confirming the reproducibility of the measurements. 

For a detailed investigation of the photodegradation byproducts by GC-MS, aliquots were collected at specific time points throughout the reaction. Each sample was prefiltered to remove any residual catalyst particles and subsequently extracted using dichloromethane as the sole organic solvent via liquid–liquid extraction. The organic phase was separated and dried over anhydrous sodium sulfate to eliminate traces of water. The dried extract was then partially concentrated under reduced pressure to enhance analyte detection, avoiding complete solvent removal. The concentrated dichloromethane solution was directly used for analysis by GC-MS. A 5 µL aliquot of the sample was injected for chromatographic separation and mass spectral identification. To ensure analytical reliability, four injections were performed for each time point: three sample replicates and one blank. Helium served as the carrier gas to ensure optimal chromatographic resolution.

### 2.6. Photocatalytic Hydrogen Production Experiments

To investigate the photocatalytic production of hydrogen by water splitting, a carefully designed experimental setup was employed. The procedure involved dispersing 50 mg of the selected catalyst in 100 mL of deionized water within a 250 mL quartz reaction vessel. To enhance electron transfer efficiency, sacrificial electron donor solutions were incorporated, consisting of sodium sulfite (Na_2_SO_3_) at a concentration of 0.03 mol/L and sodium sulfide (Na_2_S) at 0.5 mol/L [21]. The reaction system was maintained at a controlled temperature of 20 °C and purged with nitrogen (N_2_) gas for 30 min to remove dissolved oxygen and other interfering gases, ensuring an inert environment. Unlike the photocatalytic degradation experiments, which were conducted under continuous simulated solar irradiation (300–800 nm) using a dual 100 W halogen lamp system, the hydrogen evolution tests were performed under wavelength-controlled conditions. For this purpose, a UV-Vis light source was coupled with narrow-band optical filters, allowing irradiation centered at specific wavelengths (220, 320, 400, 500, 600, and 700 nm). This setup ensured that the photon flux was concentrated around each selected wavelength, allowing accurate spectral response analysis. The irradiance at the sample surface was kept constant at approximately 120 mW cm^−2^ for all wavelengths, with a uniformly irradiated area of 10 cm^2^. The experiment was performed over 2 h, allowing sufficient time for the photocatalytic splitting of water molecules and the subsequent evolution of hydrogen gas. The generated hydrogen was collected and analyzed quantitatively by gas chromatography (GC) coupled to a thermal conductivity detector (TCD). A PerkinElmer Clarus 600 chromatograph was used for precise measurement of the hydrogen concentration.

## 3. Results and Discussion

### 3.1. Characterization of the Catalysts

**Figure 1:**
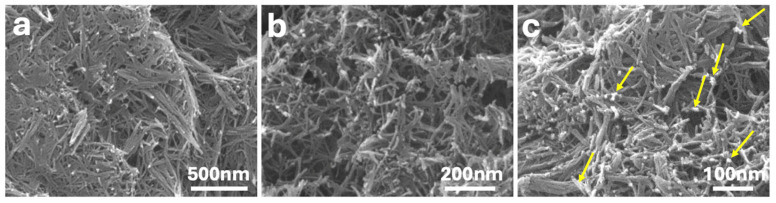
Field emission scanning electron microscopy (FESEM) image of P25-rGO at different magnifications (**a**,**b**) and 5% MoS_2_@P25-rGO (**c**). Yellow arrows in (**c**) highlight dispersed MoS_2_ nanosheets.

Figure 1 shows SEM images of the synthesized materials at increasing magnifications. The P25-rGO support (Figure 1a,b) exhibits an interconnected network of fibrous TiO_2_ structures with lengths of several hundred nanometers and diameters of less than 20 nm. These nanofibers form a porous and open framework that facilitates mass transport and light penetration. The reduced graphene oxide (rGO) component is not distinguishable at this scale due to its low contrast and highly exfoliated nature; however, it is expected to interweave throughout the fibrous TiO_2_ matrix, supporting structural cohesion and charge transport. In the MoS_2_-modified catalyst (Figure 1c), corresponding to 5 wt% MoS_2_@P25-rGO, small and dispersed bright domains can be observed along the TiO_2_ fibers (highlighted by yellow arrows), which are attributed to the localized deposition of MoS_2_ nanosheets. These features suggest a good distribution of MoS_2_ without bulk aggregation, maintaining the structural integrity of the fibrous support. This morphology agrees with reports by Gao et al. [22] and Han et al. [13], who observed similar nanosheet dispersion patterns in TiO_2_–MoS_2_–graphene composites with low MoS_2_ loadings. Although individual MoS_2_ nanosheets are not clearly resolved due to their low contrast and small size relative to the fibrous matrix, the presence of MoS_2_ in the composites is supported by complementary techniques such as XRD, Raman, and XPS, which provide clear spectroscopic and structural evidence of its successful incorporation.

**Figure 2:**
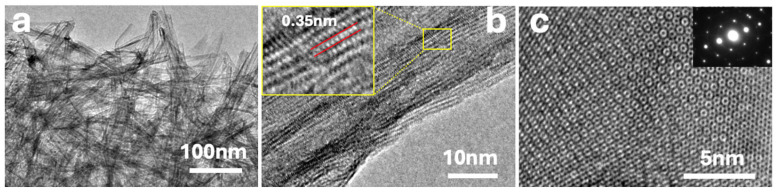
High-resolution transmission electron microscopy (HRTEM) images of the synthesized materials: (**a**) P25-rGO composite; (**b**) P25-rGO at higher magnification, with the inset highlighting lattice fringes corresponding to an interplanar spacing of approximately 0.35 nm, assigned to the (101) plane of anatase TiO_2_; and (**c**) a monolayer MoS_2_ sheet, with the inset displaying the selected area electron diffraction (SAED) pattern characteristic of hexagonal 2H-MoS_2_. This image is provided to illustrate the structural quality of the exfoliated MoS_2_ precursor and is not intended as direct evidence of its distribution within the final composite.

The different composites were also characterized by HRTEM (see Figure 2). Figure 2a shows a dense arrangement of TiO_2_ nanofibers, with rGO sheets visible as faint, translucent layers enveloping the oxide structures. Figure 2b provides a higher-resolution view of an individual TiO_2_ fiber, where lattice fringes are clearly observed. The inset highlights an interplanar spacing of ca. 0.35 nm, corresponding to the (101) plane of anatase TiO_2_, consistent with XRD analysis. Figure 2c shows an HRTEM image of a single-layer MoS_2_ nanosheet. This image corresponds to the exfoliated MoS_2_ precursor prior to its incorporation into the composite and is included to illustrate the morphology and crystalline quality of the starting material, which is consistent with that reported in MoS_2_-based heterostructures showing high HER activity [15]. The atomically resolved honeycomb pattern indicates high structural quality and confirms the presence of monolayer MoS_2_. The corresponding SAED pattern (inset) reveals a hexagonal diffraction arrangement, characteristic of the 2H-phase of MoS_2_. The clear spots and absence of diffuse rings confirm high crystallinity and minimal structural defects. Although HRTEM images of the full composite are not shown here, the incorporation of MoS_2_ into the P25–rGO matrix is strongly supported by structural and spectroscopic evidence; in particular, the presence of MoS_2_ peaks in XRD, the detection of MoS_2_-specific bands in Raman spectra, and the identification of Mo and S oxidation states in XPS collectively validate the successful formation of the ternary nanocomposite.

**Figure 3:**
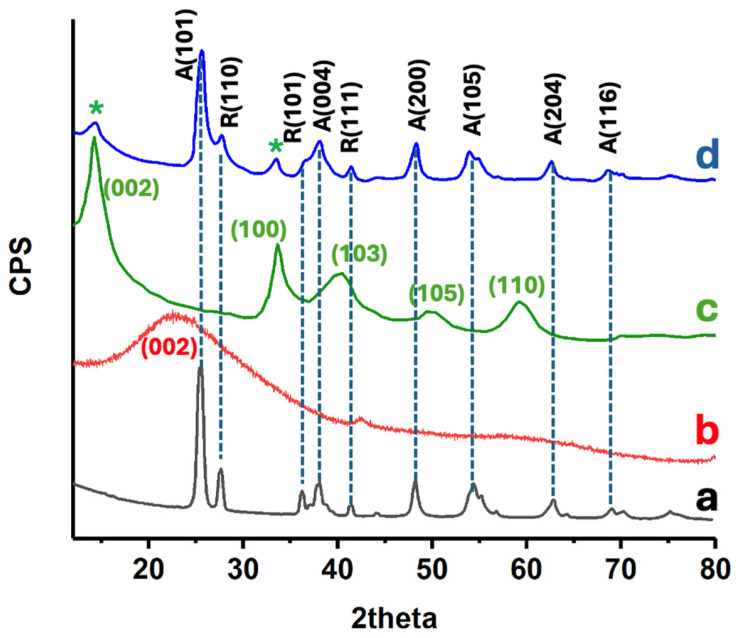
XRD patterns of commercial TiO_2_-P25 (**a**), rGO (**b**), exfoliated MoS_2_ (**c**), and 5% MoS_2_@P25-rGO (**d**).

The crystalline phase composition of the prepared catalysts was examined by XRD (see Figure 3). Pure P25 TiO_2_ exhibits the most characteristic reflections of anatase TiO_2_ at 25.5°, 38°, 48.2°, 54.4°, assigned to the (101), (004), (200), (105) planes of anatase, along with a rutile peak at ca. 27.7°, consistent with its well-known mixed-phase composition [22]. The relative anatase-to-rutile ratio was estimated using the Spurr and Myers method [23], based on the intensity ratio of the anatase (101) and rutile (110) peaks. The calculated ratio was approximately 80:20, in agreement with the nominal composition of commercial P25. Importantly, this mixed-phase structure was retained after the incorporation of rGO and MoS_2_, as no significant changes were observed in peak position or intensity. This indicates that the crystalline integrity of TiO_2_ and its phase composition were preserved throughout the synthesis. Such stability is advantageous, as the coexistence of anatase and rutile is known to enhance charge separation in photocatalysis. The TiO_2_-rGO composite shows a virtually identical diffraction pattern to P25, indicating that the TiO_2_ retained its crystalline structure after the graphene incorporation. Notably, no distinct new peaks attributable to graphene are observed; any potential (002) graphitic peak (~23°) is broadened or overlapped by the strong TiO_2_ (101) peak [22]. This is expected given the low loading and exfoliated nature of rGO, which lacks long-range order in stacking. Upon adding MoS_2_, the composite XRD patterns still predominantly display TiO_2_ reflections, but new low-angle peaks appear. In particular, a faint diffraction peak appears around 13–14° in the 5% MoS_2_@TiO_2_-rGO sample (see asterisk), corresponding to the (002) basal plane of hexagonal MoS_2_ [22]. An additional minor peak at ca. 33° can be discerned, matching the (100) plane of MoS_2_ [24] (see asterisk). The presence of these MoS_2_ reflections confirms the successful incorporation of crystalline MoS_2_ into the TiO_2_-rGO matrix. These observations are in agreement with previously reported diffraction patterns for TiO_2_–MoS_2_ composites, where similar low-angle reflections for MoS_2_ were observed without altering the TiO_2_ lattice [22,24]. Importantly, no significant shifts in the TiO_2_ peak positions are detected upon MoS_2_ or rGO addition, suggesting that Mo and S are not substituting into the TiO_2_ lattice, but rather that MoS_2_ and rGO form an intimate heterostructure on the TiO_2_ surface. The combination of TiO_2_ and MoS_2_ diffraction features, with no extra impurity phases, evidences the formation of the intended composite.

**Figure 4:**
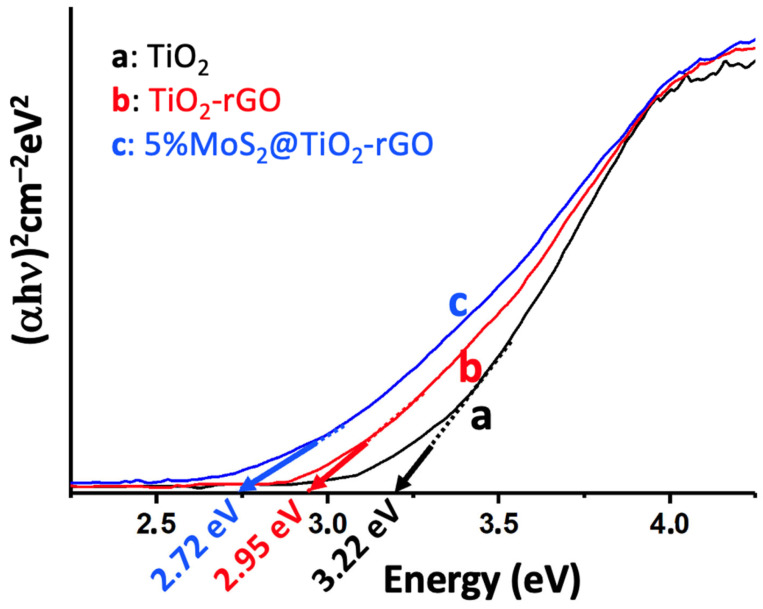
Tauc plots of (αhν)^2^ versus energy (eV) and determination of the bandgap energy of TiO_2_ (P25) (**a**), TiO_2_-rGO (**b**), and 5% MoS_2_@TiO_2_-rGO (**c**).

UV-Vis DRS was used to assess the optical absorption properties and bandgap energies of the catalysts (Figure 4). Pristine P25 TiO_2_ shows a strong absorption edge in the UV region (ca. 390 nm), corresponding to a bandgap of about 3.22 eV (consistent with anatase TiO_2_) [25]. The incorporation of rGO extends the absorption into the visible range (the TiO_2_-rGO sample appears darker) with a red-shifted absorption edge. Tauc plot analysis (Figure 4) indicates a reduced bandgap of ~2.95 eV for TiO_2_-rGO, implying that the introduction of rGO facilitates visible-light absorption. This bandgap narrowing can be attributed to the electronic interaction between TiO_2_ and the conductive rGO, which may introduce mid-gap states and promote the formation of an adsorption tail in the band structure. Upon loading 5% MoS_2_ onto TiO_2_-rGO, the absorption edge shifts further into the visible (up to ca. 455–460 nm), yielding an estimated optical bandgap of ca. 2.72 eV for the 5% MoS_2_@TiO_2_-rGO composite [25]. The progressive red shift in the absorption onset from 3.22 eV (TiO_2_) to 2.72 eV (MoS_2_@TiO_2_-rGO) confirms that the synergy of rGO and MoS_2_ effectively extends the light-harvesting range of TiO_2_ into the visible spectrum. This behavior is consistent with the MoS_2_ acting as a narrow-bandgap sensitizer (2H-MoS_2_ has a much smaller bandgap of ~1.2–1.8 eV) and the rGO acting as a photosensitizer and electron conduit [25]. The black-colored MoS_2_ nanosheets strongly absorb visible light, and when coupled with TiO_2_, enable the heterostructure to use a greater portion of the solar spectrum [25]. In addition, intimate contact between TiO_2_ and MoS_2_ (and rGO) can create sub-bandgap states or band bending at the interface, further contributing to the observed bandgap reduction [26,27]. The enhanced visible-light absorption, as evidenced by DRS, directly correlates with improved photocatalytic activity under solar irradiation; by harvesting more photons in the visible range, the MoS_2_@TiO_2_-rGO catalyst can generate more charge carriers for pollutant degradation and H_2_ evolution compared to pure TiO_2_ [28]. This progressive bandgap narrowing matches trends observed in other ternary photocatalysts combining rGO and MoS_2_ with TiO_2_, as shown in works by Tien and Chen [29] and Panchal et al. [14], where red shifts were likewise attributed to interfacial band bending and sensitization effects.

**Figure 5:**
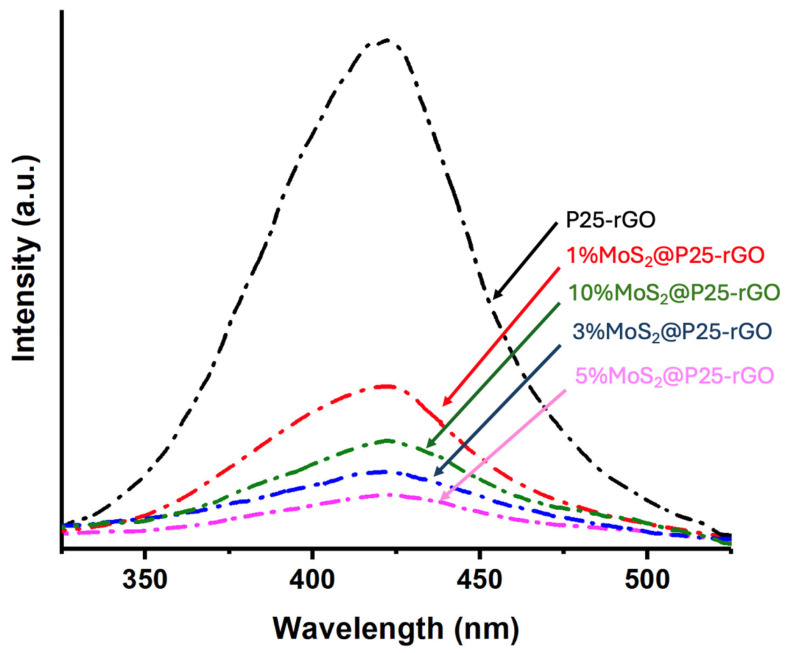
PL spectra of P25-rGO, 1% MoS_2_@P25-rGO, 3% MoS_2_@P25-rGO, 5% MoS_2_@P25-rGO, and 10% MoS_2_@P25-rGO.

Photoluminescence spectroscopy was used to investigate the recombination behavior of photogenerated charge carriers in the photocatalysts. Figure 5 displays the room-temperature PL emission spectra (λ_exc_ = 380 nm) for P25-rGO and its composites containing different MoS_2_ loadings (1%, 3%, 5%, and 10%). The P25-rGO sample exhibits a strong and broad emission band in the UV-visible range, reflecting a high rate of radiative recombination of electron–hole pairs in the absence of additional charge separation pathways [30]. Upon incorporation of MoS_2_, the PL intensity generally decreases, indicating improved charge separation due to the synergistic effects of MoS_2_ and rGO [29]. The quenching trend follows the following order: P25-rGO > 1% MoS_2_@P25-rGO > 10% MoS_2_@P25-rGO > 3% MoS_2_@P25-rGO > 5% MoS_2_@P25-rGO, with the 5% MoS_2_@P25-rGO composite showing the lowest PL intensity among all tested materials. Interestingly, the composite with 10% MoS_2_ exhibits a higher PL intensity than those with 3% and 5%, suggesting that excessive MoS_2_ content may not be beneficial. This could be due to the agglomeration of MoS_2_ layers or shielding effects that interfere with light absorption and charge transfer processes. Therefore, beyond an optimal loading, MoS_2_ may hinder rather than enhance photocatalytic performance [29]. Overall, the PL quenching confirms that moderate MoS_2_ incorporation enhances charge carrier separation, while excessive loading could counteract this benefit. The significant PL reduction observed in 5% MoS_2_@P25-rGO points to an optimal interfacial configuration among TiO_2_, rGO, and MoS_2_ that favors efficient charge extraction and transport [24,29,30]. Similar PL suppression patterns have been reported by Zhang et al. [30] and Quan et al. [31], who attributed them to the combined role of rGO as an electron sink and of MoS_2_ as an active charge-transfer co-catalyst. This agreement supports the reliability of the observed recombination trends in our system. These results are consistent with the photocatalytic activity trends, as will be discussed in a later section, where the 5% MoS_2_ composite also displayed the highest performance in both malathion degradation and hydrogen evolution, confirming that suppressed electron–hole recombination is a key factor in the enhanced reactivity of these ternary composites.

**Figure 6:**
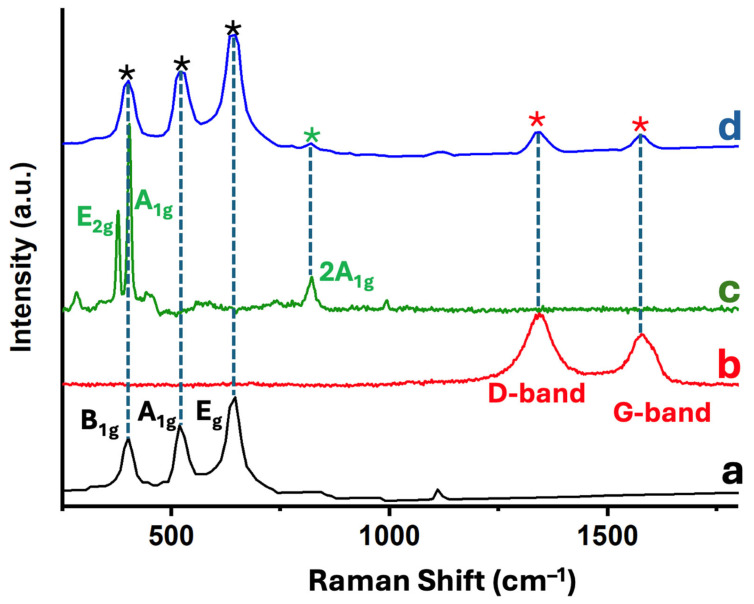
Raman spectra of the TiO_2_-P25 (**a**), rGO (**b**), exfoliated MoS_2_ (**c**), and 5% MoS_2_@TiO_2_-rGO (**d**). The black, red and green asterisks represent peaks assigned to TiO_2_-P25, rGO and MoS_2_, respectively.

Raman spectroscopy was employed to investigate the structural features and component interactions within the 5% MoS_2_@TiO_2_-rGO photocatalyst. Figure 6 displays the Raman spectra of individual and composite materials: (a) TiO_2_-P25, (b) rGO, (c) MoS_2_, and (d) the ternary nanocomposite 5% MoS_2_@TiO_2_-rGO. In the spectrum of pristine TiO_2_-P25 (Figure 6a), three characteristic vibrational modes of the anatase phase are clearly observed at approximately 398 cm^−1^ (B_1g_), 518 cm^−1^ (A_1g_ + B_1g_), and 640 cm^−1^ (E_g_) [22]. The spectrum of rGO (Figure 6b) exhibits two prominent and broad peaks centered at ca. 1345 cm^−1^ (D band) and 1590 cm^−1^ (G band). The G band arises from the E_2g_ vibrational mode of sp^2^ carbon atoms (graphitic domains), while the D band originates from defect-activated breathing modes in disordered sp^2^ structures [22]. The observed intensity ratio (I_D_/I_G_ ≈ 0.8–1.0) suggests a partially reduced graphene oxide with residual structural defects and oxygenated functionalities, which is an expected outcome of mild reduction protocols. In the MoS_2_ spectrum (Figure 6c), the in-plane E_2_g^1^ mode (ca. 379 cm^−1^) and the out-of-plane A_1_g mode (ca. 404 cm^−1^) characteristic of the 2H phase of MoS_2_ are clearly observed [30]. The band separation (~25 cm^−1^) is consistent with that of few-layer MoS_2_, as larger separations are typical in thinner nanosheets due to decreased interlayer interactions [24]. A weak overtone near 990 cm^−1^, attributed to the 2A_1_g mode, further supports the presence of multilayer characteristics. The composite 5% MoS_2_@TiO_2_-rGO (Figure 6d) presents vibrational features from all three components. The anatase TiO_2_ bands (black asterisks), the rGO D and G bands (red asterisks), and the MoS_2_ peaks (green asterisks) are all clearly visible, confirming the coexistence of each constituent in the hybrid structure [30]. Importantly, no new bands or significant peak shifts are observed, suggesting that no undesirable side reactions (e.g., Mo oxidation, Ti–C bonding, or carbide formation) occurred during synthesis. These results validate the structural integrity of the ternary composite and the successful assembly of TiO_2_, rGO, and MoS_2_ without phase degradation. The presence of well-defined and distinct vibrational signatures from each component further implies favorable interfacial contact, which may facilitate charge separation and transport, critical factors in enhancing photocatalytic activity.

The specific surface areas of the synthesized materials were determined via nitrogen adsorption–desorption measurements using the Brunauer–Emmett–Teller (BET) method. As summarized in Table S1, the commercial P25 TiO_2_ sample exhibited a surface area of 48 m^2^/g, consistent with its well-established properties. The measured BET surface area of the reduced graphene oxide (rGO) used in this study was 429 m^2^/g, which aligns with values typically reported for exfoliated rGO synthesized under mild reduction conditions [32]. Upon incorporation of reduced graphene oxide (rGO), the surface area increased substantially to 483 m^2^/g, reflecting the textural contribution of rGO sheets, which help prevent TiO_2_ agglomeration and promote a more open porous structure. Further addition of exfoliated MoS_2_ led to a progressive increase in surface area, with values of 492, 496, 503, and 521 m^2^/g for the composites containing 1%, 3%, 5%, and 10% MoS_2_, respectively. These results demonstrate that the inclusion of rGO is the main contributor to the enhancement in surface area relative to bare TiO_2_, while incremental increases are further achieved through MoS_2_ nanosheet incorporation. This trend suggests that the introduction of layered MoS_2_ contributes to additional mesoporosity and helps maintain a high surface-to-volume ratio in the hybrid system. Interestingly, however, as will be discussed in subsequent sections, the composite with 10% MoS_2_, despite having the highest BET surface area, exhibited inferior photocatalytic performance in both malathion degradation and hydrogen evolution. This apparent contradiction can be explained by considering that, beyond a critical MoS_2_ loading, excessive nanosheet accumulation may cause partial shielding of the TiO_2_ surface, hinder interfacial charge transfer, or create electron–hole recombination centers. Although surface area is a relevant parameter in catalysis, it is not the sole determinant of photocatalytic performance. In our case, the 10% MoS_2_ composite likely exhibits poor charge carrier mobility due to MoS_2_ restacking or dense coverage, which can reduce photon penetration and suppress the formation of effective heterojunctions with TiO_2_ and rGO. This interpretation is supported by the increased PL intensity and reduced activity observed for this sample. This highlights that surface area alone is not the determining factor for photocatalytic efficiency. This finding is consistent with prior observations by Gao et al. [22] and Chang et al. [25], which indicate that higher surface areas induced by excessive MoS_2_ lead to decreased photocatalytic performance due to restacking and site-blocking phenomena. At higher MoS_2_ contents, excessive coverage or restacking of MoS_2_ layers may hinder light absorption or block active sites, disrupting the optimal heterojunction structure necessary for efficient charge separation and transfer [33].

**Figure 7:**
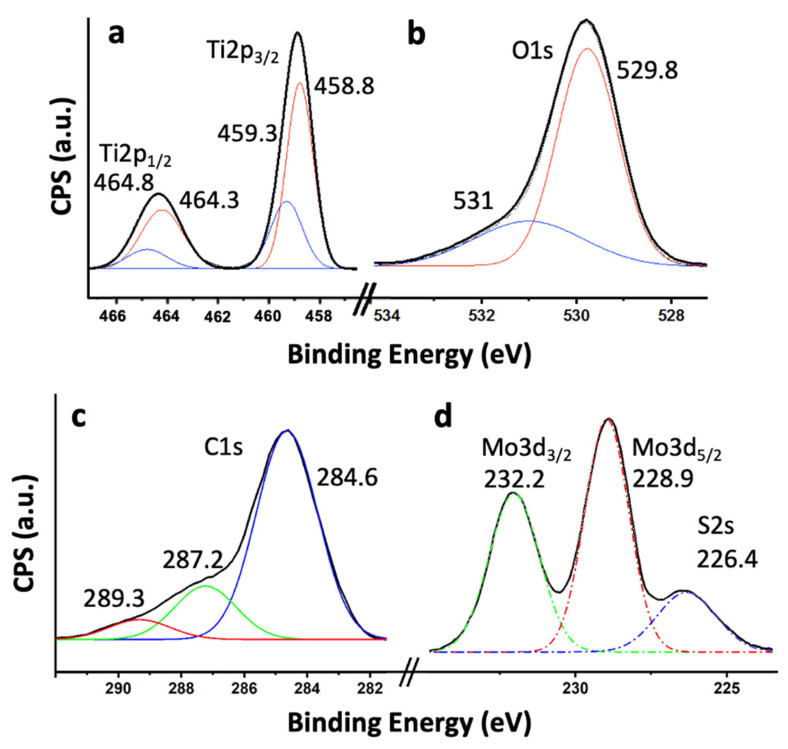
XPS core level spectra for 5% MoS_2_@TiO_2_-rGO: Ti2p (**a**), O1s (**b**), C1s (**c**), and Mo3d-S2s (**d**).

To investigate the surface chemical composition and oxidation states of the elements present in the 5% MoS_2_@TiO_2_-rGO composite, high-resolution X-ray photoelectron spectroscopy (XPS) analyses were performed (Figure 7). The spectra confirm the presence of all key elements: Ti, O, C, Mo, and S. As shown in Figure 7a, the high-resolution Ti 2p spectrum reveals two well-defined peaks at 458.8 eV and 464.3 eV, corresponding to Ti 2p_3_/_2_ and Ti 2p_1_/_2_, respectively, which are characteristic of Ti^4^^+^ in TiO_2_ [34,35]. A minor shoulder at ca. 459.3 eV may indicate surface heterogeneity or electronic interactions with MoS_2_ or rGO [36], but no significant signal is observed at lower binding energies to suggest the presence of Ti^3^^+^ species, confirming that the TiO_2_ structure remains predominantly in the fully oxidized state [34]. The O 1s spectrum (Figure 7b) shows a major peak at 529.8 eV attributed to lattice oxygen (Ti–O–Ti) and a secondary component at 531.0 eV, which corresponds to surface hydroxyl groups, adsorbed water, or oxygenated species on rGO [37]. These surface oxygen functionalities are often associated with enhanced photocatalytic activity, as they can facilitate charge separation and radical formation [37]. The C 1s spectrum (Figure 7c) displays a dominant signal at 284.6 eV due to sp^2^-hybridized carbon atoms in the graphene lattice (C=C), along with minor peaks at 287.2 eV and 289.3 eV that can be assigned to carbonyl (C=O) and carboxyl (O–C=O) groups, respectively [32,38]. The relatively low intensity of these oxidized carbon species confirms the successful partial reduction of graphene oxide to rGO, while the residual functional groups are beneficial for improving interfacial bonding and electron transfer between components [39]. The Mo 3d spectrum (Figure 7d) exhibits two main peaks, located at 228.9 eV (Mo 3d_5_/_2_) and 232.2 eV (Mo 3d_3_/_2_), characteristic of Mo^4^^+^ in MoS_2_ [34,40]. No additional peaks are detected in the higher binding energy range (233–235 eV), ruling out the presence of significant amounts of oxidized Mo^6+^ species such as MoO_3_ [40]. In the same region, a broad feature at 226.4 eV is assigned to the S 2s signal [41], further supporting the existence of sulfide species (S^2−^) in the MoS_2_ lattice [34,41]. Altogether, the XPS results confirm the integration of TiO_2_, MoS_2_, and rGO into a ternary heterostructure with minimal chemical perturbation and strong interfacial interactions. The preservation of the oxidation states of Ti^4+^ and Mo^4+^, along with the partial reduction of rGO, is consistent with the enhanced photocatalytic behavior observed in degradation and hydrogen evolution experiments. This electronic structure is comparable to that reported for optimized TiO_2_–MoS_2_–graphene hybrids exhibiting enhanced hydrogen evolution activity [14,34], reinforcing the proposed synergistic interaction between the components.

### 3.2. Photocatalytic Degradation of Malathion

To establish the optimal reaction conditions, a series of preliminary experiments were carried out using the most active material, 5% MoS_2_@TiO_2_-rGO, as the reference (see Figure S1). These studies focused on evaluating the influence of key operational parameters, such as catalyst loading, the initial pH of the solution, and the presence or absence of irradiation and oxygen, on the degradation of malathion. The outcomes not only allowed us to determine the ideal experimental conditions for maximum photocatalytic efficiency but also served to confirm the photocatalytic origin of the degradation process through control experiments. These optimized parameters were subsequently applied in the evaluation of the remaining catalysts to ensure consistent and comparable performance assessments. Figure S1a shows the effect of catalyst loading (from 0.4 to 1.8 g/L) on the photodegradation efficiency of malathion after 2 h of UV-visible irradiation. An increase in catalyst loading led to improved degradation up to an optimal concentration of 1.0 g/L, where the degradation reached nearly 100%. This enhancement is attributed to the increased number of active sites and photon absorption capacity. However, beyond this concentration, the degradation efficiency decreased significantly. At 1.6 and 1.8 g/L, degradation dropped to around 65% and 50%, respectively. This decline is likely due to increased turbidity and light scattering at higher catalyst concentrations, which reduce light penetration and active photon flux within the suspension. Figure S1b shows the influence of the solution pH on photocatalytic degradation efficiency. Experiments were performed over a pH range of 4 to 10, keeping all other conditions constant. The photocatalytic activity showed a marked dependence on pH, with maximum degradation (ca. 100%) occurring at neutral to slightly acidic conditions (pH 6–7). Below this range, especially at pH 4, degradation efficiency decreased sharply (~60%), likely due to reduced malathion adsorption or catalyst surface protonation. In alkaline media (pH > 8), the degradation also decreased, possibly due to hydroxide ion competition or destabilization of reactive oxygen species. These results suggest that the surface charge of the photocatalyst and the speciation of malathion both influence the reaction kinetics, and that near-neutral conditions are ideal for optimal degradation. To confirm the photocatalytic nature of the malathion degradation process, a series of control tests were conducted (Figure S1c). These included (i) photolysis (irradiation without catalyst), (ii) catalysis (catalyst in the dark), and (iii) photocatalysis under anoxic conditions (an argon-purged system). The results clearly show that significant degradation occurred only under full photocatalytic conditions (light + catalyst + air), where the malathion concentration dropped steadily over time, reaching almost complete mineralization within 120 min. In contrast, all control conditions showed minimal activity: photolysis and catalysis resulted in only minor losses (<15%), and the anoxic photocatalytic test demonstrated reduced efficiency, highlighting the essential role of dissolved oxygen as an electron acceptor in the generation of reactive oxygen species (ROS).

**Figure 8:**
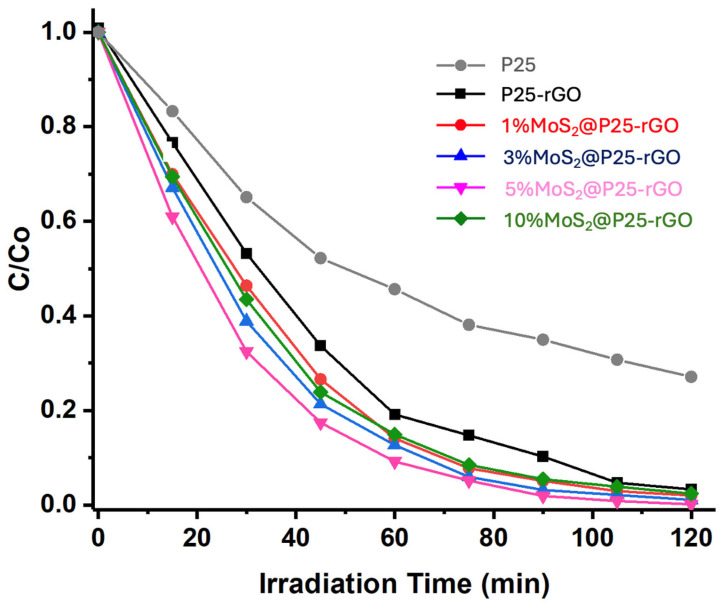
Malathion degradation profiles for pristine P25, P25-rGO, and MoS_2_@P25-rGO composites under simulated solar irradiation. Each data point represents the average of three independent experiments. Error bars were omitted for clarity, as the standard deviation was below 4% and the overlap of multiple curves impeded visual interpretation.

The photocatalytic activity of the synthesized materials was evaluated under UV-visible irradiation. Two complementary light sources were employed in this study, depending on the experimental objective. For general photocatalytic degradation and hydrogen evolution experiments, a dual 100 W halogen lamp system (Philips, warm white) was used, providing a total irradiance of approximately 6300 lux, simulating AM 1.5 solar light conditions [20]. AM 1.5 refers to the standard terrestrial solar spectrum at mid-latitudes, where sunlight passes through 1.5 times the atmosphere relative to its path at zenith, corresponding to typical daylight conditions [42]. In contrast, for wavelength-dependent hydrogen production studies, monochromatic light was obtained using optical bandpass filters to isolate specific wavelengths (e.g., 220, 320, 400 nm), allowing selective analysis of the photoresponse at different energy intervals. These experimental configurations are described in detail in Section 2.5. The performance of the different catalysts—P25-rGO and MoS_2_-modified composites with 1%, 3%, 5%, and 10% MoS_2_—was compared under previously optimized reaction conditions (1.0 g/L catalyst loading and pH 7). As shown in Figure 8, the pristine P25 sample exhibited the lowest degradation efficiency, highlighting its limited activity under visible-light-rich conditions and establishing a baseline for comparison. In contrast, all MoS_2_-containing composites outperformed both pristine P25 and the P25-rGO sample, demonstrating the beneficial effect of MoS_2_ addition. Among the tested materials, 5% MoS_2_@P25-rGO exhibited the highest degradation rate, achieving near-complete removal of malathion within 120 min. This enhanced activity is attributed to the synergistic interaction among TiO_2_, rGO, and MoS_2_, which promotes charge separation and broadens light absorption. The 3% MoS_2_ and 1% MoS_2_ composites also showed significant improvements compared to the P25-rGO, but to a lesser extent. Interestingly, the 10% MoS_2_@P25-rGO catalyst exhibited slightly lower activity than the 3% and 5% counterparts, likely due to excessive MoS_2_ loading that can shield the active surface or induce recombination centers, corroborating the previously discussed BET and PL results. Despite having the highest measured surface area (521 m^2^/g), the 10% MoS_2_ composite showed limited photocatalytic efficiency. This suggests that excessive MoS_2_ may form agglomerates or restacked layers that reduce the effectiveness of photon absorption and hinder charge transfer pathways within the heterostructure. As PL spectroscopy confirms, recombination becomes more pronounced with higher MoS_2_ content, offsetting the benefit of additional surface area. These results indicate that achieving a balance between surface accessibility and interfacial charge dynamics is crucial, with 5% MoS_2_ representing the optimal composition in this system.

To better understand the degradation mechanism of malathion using the 5% MoS_2_@P25-rGO composite, a series of scavenger experiments was conducted to identify the main reactive species involved (see Figure S2). The addition of 1,4-benzoquinone (BQ), a selective quencher of superoxide radicals (·O_2_^−^) [43], resulted in a pronounced decrease in degradation efficiency, strongly suggesting that ·O_2_^−^ species play a central role in the photocatalytic process [43]. In contrast, the use of EDTA-Na_2_, a hole (h^+^) scavenger [44], led to negligible inhibition, indicating that direct oxidation by photogenerated holes is not the primary degradation pathway [44]. Similarly, the addition of tert-butanol (t-BuOH), a hydroxyl radical (·OH) scavenger [45,46], caused only moderate suppression, pointing to a secondary contribution of ·OH radicals [45,46]. These findings are consistent with a mechanism in which photoexcited electrons, generated upon UV-visible irradiation of TiO_2_, are efficiently transferred to MoS_2_ and/or rGO, reducing adsorbed O_2_ molecules to form superoxide radicals. The layered structure and intimate contact among TiO_2_, MoS_2_, and rGO facilitate efficient charge separation and migration across the heterostructure, possibly through a Type-II or Z-scheme charge transfer mechanism. MoS_2_, with its suitable conduction band position, acts as an electron acceptor and stabilizer, while rGO provides a rapid electron transport pathway [15,47]. The result is enhanced generation of ·O_2_^−^ species, which act as the dominant oxidizing agents responsible for the breakdown of malathion.

**Figure 9:**
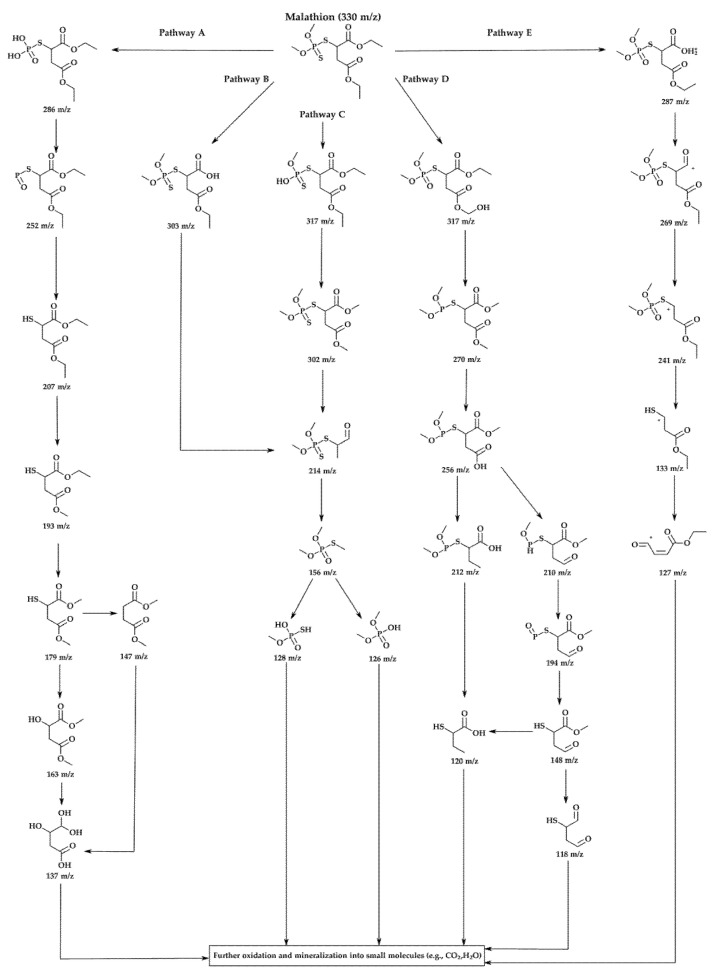
Proposed photocatalytic degradation pathways of malathion (*m*/*z* 330) under UV-visible irradiation using the 5% MoS_2_@P25-rGO catalyst, based on GC-MS analysis. Five main degradation routes (Pathways A–E) were identified, involving hydrolysis, desulfuration, C–O and P–S bond cleavage, oxidative demethylation, and ring-opening reactions.

A detailed GC-MS analysis was conducted to elucidate the photocatalytic degradation pathway of malathion under UV-visible irradiation using the most active catalyst, 5% MoS_2_@P25-rGO (Figure 9). Reaction aliquots were collected at different irradiation times and analyzed to identify intermediate products based on their mass-to-charge (*m*/*z*) ratios. The parent compound, malathion (*m*/*z* = 330), was progressively decomposed through a sequence of hydrolytic and oxidative transformations. Five main degradation pathways (A–E) were proposed based on the detected fragments and their temporal evolution, as illustrated in Figure 9. In Pathway A, the hydrolysis of ester bonds and ring opening resulted in the formation of lower-mass products [48]. Pathways B and C involve oxidative desulfuration and P–S bond cleavage, producing fragments such as *m*/*z* 303, 302, 214, and 156 [49,50,51,52]. Pathway D comprises further oxidation and sulfur removal, generating species at *m*/*z* 317, 270, and 256 [48], while Pathway E involves oxidative demethylation and side-chain fragmentation, yielding intermediate ions like *m*/*z* 287, 241, and 133 [53]. The presence of low-molecular-weight fragments (*m*/*z* 128, 126, 137) indicates the occurrence of advanced oxidation processes, suggesting partial mineralization of malathion into CO_2_ and H_2_O, consistent with the mineralization trends observed in other TiO_2_-based systems. Importantly, the formation of intermediates such as *m*/*z* 214 and 156 supports the predominant role of superoxide radicals (·O_2_^−^) as oxidative agents, consistent with the radical trapping experiments discussed previously [54,55]. The heterostructure of TiO_2_, MoS_2_, and rGO favors efficient charge separation and facilitates electron transfer to molecular oxygen, sustaining the generation of reactive oxygen species (ROS). MoS_2_, due to its narrow bandgap and appropriate conduction band alignment, acts as an effective electron sink, while rGO enhances electron transport and surface dispersion. This synergistic configuration promotes a Z-scheme or Type II-like mechanism that enhances photoinduced redox activity [56]. Altogether, the results demonstrate that the 5% MoS_2_@P25-rGO catalyst enables efficient and multi-step degradation of malathion via concurrent hydrolytic and oxidative pathways, ultimately leading to detoxification of the pollutant and partial mineralization under mild conditions.

To ensure the practical viability of the developed photocatalysts, long-term operational stability and reusability were also evaluated. In this context, a recyclability study was conducted using the most active material, 5% MoS_2_@P25-rGO, to assess its performance over successive degradation cycles. As shown in Figure S3, the photocatalyst maintained nearly constant activity throughout 10 consecutive runs, with only a slight decline of approximately 4.7% in degradation efficiency. This stability underscores the structural robustness and chemical durability of the MoS_2_-rGO-TiO_2_ heterojunction, confirming its suitability for repeated use in aqueous photocatalytic systems under UV-visible light irradiation.

**Figure 10:**
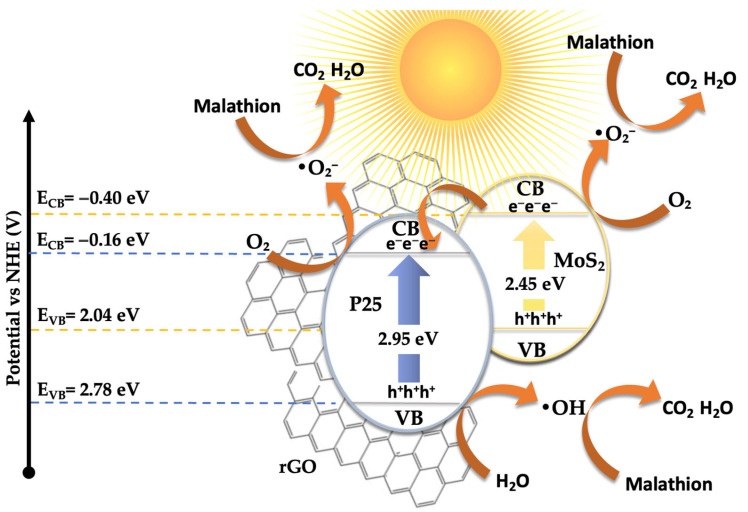
Schematic diagram of the proposed mechanism for the photodegradation of malathion, using the 5% MoS_2_@P25-rGO catalyst under irradiation.

Based on all the results presented above, a plausible mechanism for the photocatalytic degradation of malathion has been proposed, as illustrated in Figure 10. The electronic band structure and the migration direction of photogenerated charge carriers were estimated using the Mulliken electronegativity theory [57,58,59,60]. This approach, originally introduced by R. S. Mulliken [60], relates the absolute electronegativity (χ) of a material to its ability to attract electrons. In this context, χ is defined as the arithmetic mean of the first ionization energy and the electron affinity of the compound and reflects its electron-attracting strength. For compound semiconductors such as TiO_2_ and MoS_2_, χ values are typically derived from weighted averages of their constituent atomic electronegativities and are available in the literature [60]. The band edge potentials were calculated using the following equations [57]:

E_CB_ = X − E_C_ − 0.5E_g_ (1)

E_VB_ = E_CB_ + E_g_ (2)

where E_CB_ and E_VB_ are the conduction and valence band edge potentials (in eV), Eg is the bandgap energy of the semiconductor (determined from UV-Vis DRS), E_C_ is the energy of free electrons on the hydrogen scale (taken as 4.50 eV) [61], and χ is the absolute electronegativity of the semiconductor compound, estimated from tabulated values.

The calculated electronic parameters used for this estimation are summarized in Table 1. These include the absolute electronegativity (χ), optical bandgap (Eg), and the resulting conduction and valence band edge potentials (E_CB_ and E_VB_) for the main semiconducting components in the composite system.

Based on this model, the calculated band edge positions for P25-rGO are E_CB_ = −0.165 eV and E_VB_ = +2.785 eV, while for MoS_2_, they are E_CB_ = −0.405 eV and E_VB_ = +2.045 eV. Under visible light irradiation, TiO_2_ (P25) is largely inactive due to its wide bandgap (~3.2 eV) [62]. However, MoS_2_ and rGO, with narrower bandgaps, can absorb visible photons and become photoexcited [63]. In the case of MoS_2_, visible light promotes electrons from the valence band to the conduction band, leaving behind holes. These photoexcited electrons, due to the more negative conduction band of MoS_2_ (–0.405 eV) relative to P25-rGO (–0.165 eV), can transfer to the TiO_2_–rGO interface, where they are readily scavenged by molecular oxygen dissolved in the medium. This reduction leads to the formation of superoxide radicals (·O_2_^−^), which are highly reactive and capable of oxidizing malathion. Simultaneously, holes remaining in the MoS_2_ and photoinduced holes in the rGO may weakly contribute to oxidation, although scavenger experiments indicate that their role is secondary (see Figure S2). Instead, hydroxyl radicals (·OH), generated from water or hydroxide oxidation by valence band holes in the TiO_2_, provide an additional oxidative pathway. The high surface area of rGO facilitates these processes by providing a large number of adsorption and reaction sites while also improving charge mobility and suppressing recombination via rapid electron conduction [64]. The dominant degradation route, as supported by radical quenching experiments and GC-MS analysis, is thus initiated by ·O_2_^−^ radicals attacking the ester and phosphorothioate bonds in malathion, leading to a stepwise oxidative fragmentation into less toxic and lower-molecular-weight intermediates. This mechanism is fully consistent with the observed suppression of activity upon the addition of 1,4-benzoquinone (a ·O_2_^−^ scavenger), as well as with the enhanced photocatalytic activity shown by the 5% MoS_2_@P25-rGO composite compared to binary or unmodified systems. The proposed mechanism involves a type-II heterojunction [65], in which MoS_2_ and rGO sensitize the composite to visible light [14], and the hierarchical structure promotes directional charge transfer from MoS_2_ to TiO_2_–rGO [31]. This configuration enables the generation of reactive oxygen species—mainly superoxide and, to a lesser extent, hydroxyl radicals—which drive the oxidative degradation of malathion under solar-like irradiation conditions.

### 3.3. Photocatalytic Hydrogen Production

As performed for the malathion photodegradation studies, a similar approach was employed to determine the optimal conditions for photocatalytic hydrogen production (Figure S4). The influence of catalyst loading on the photocatalytic hydrogen production performance of the most active nanocomposite, 5% MoS_2_@P25-rGO, was first investigated (Figure S4a). The hydrogen evolution rate increased with catalyst concentration up to an optimal loading of 1.0 g L^−1^, reaching a maximum yield of nearly 6000 µmol g^−1^ h^−1^. Beyond this point, the activity decreased, likely due to excessive light scattering, increased turbidity, and the agglomeration of photocatalyst particles, which limit light penetration and reduce the number of accessible active sites. At lower catalyst dosages, the lower availability of surface-active regions similarly limits the overall rate of hydrogen generation. The effect of solution pH on hydrogen production was subsequently evaluated (Figure S4b). The system exhibited optimal performance under neutral conditions (pH = 7), where both charge carrier separation and proton availability are favorably balanced. In strongly acidic environments (pH = 4), the excessive concentration of H^+^ ions can hinder charge mobility and promote recombination. Conversely, under alkaline conditions (pH = 10), the reduced proton concentration limits the supply of reactants necessary for H_2_ evolution, leading to a significant drop in photocatalytic efficiency [66]. To confirm the photocatalytic nature of the observed hydrogen generation, control experiments were conducted under different conditions (Figure S4c). Negligible H_2_ evolution was detected in the absence of either the catalyst or light, confirming that both components are essential for the reaction to proceed. These results validate that the process is strictly photo-driven and demonstrate the synergy among MoS_2_, TiO_2_, and rGO in facilitating efficient light-induced hydrogen evolution.

To further assess the photocatalytic performance of the optimized 5% MoS_2_@P25-rGO composite, the apparent quantum efficiency (AQE) was calculated under monochromatic irradiation at 500 nm. The AQE was determined using the following standard expression:

$$AQE\left(\%\right)=\frac{2\times mol\;of\;H_{2}\times N_{A}}{Incident\;photons}\times 100$$

The experiment was conducted using 50 mg of catalyst irradiated over 10 cm^2^ for 2 h at 500 nm, with a light intensity of 120 mW·cm^−2^. Under these conditions, the total hydrogen evolution was 600 µmol·g^−1^·h^−1^, resulting in an AQE of approximately 3.33%. The detailed calculation steps, including photon flux estimation and hydrogen quantification, are provided in the Supplementary Information (Table S2). This value is consistent with previously reported ternary TiO_2_-based systems incorporating MoS_2_ and rGO and supports the efficient utilization of visible photons in the hydrogen evolution process [67,68].

**Figure 11:**
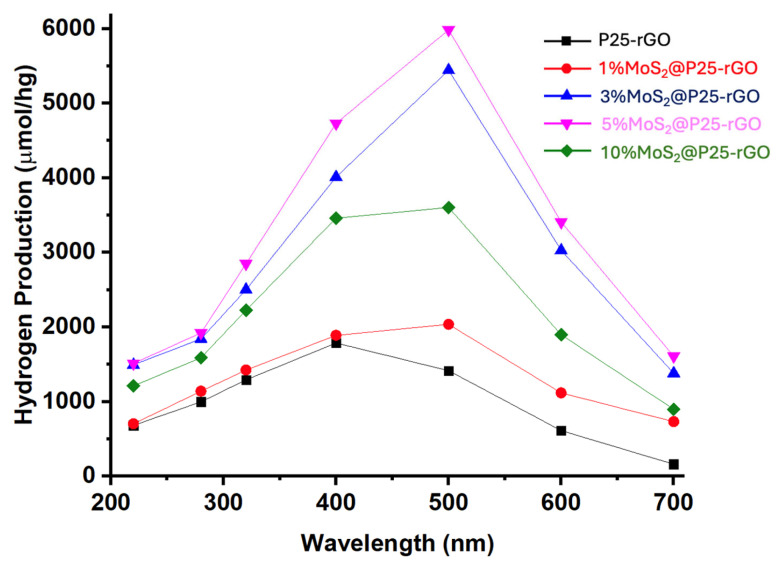
Hydrogen production profiles of the synthesized catalysts under irradiation at different wavelengths. All experiments were performed in triplicate. Although data variability was consistently below 5%, error bars were omitted to preserve visual clarity due to the overlap of multiple data series.

Figure 11 shows the wavelength-dependent hydrogen production profiles, clearly highlighting the superior photocatalytic activity of the MoS_2_-modified P25-rGO composites relative to the unmodified P25-rGO system. As a function of the incident photon energy, all catalysts show enhanced activity within the 300–500 nm range, where the absorption of UV and visible light is most efficient. Among the materials studied, the 5% MoS_2_@P25-rGO nanocomposite exhibits the highest hydrogen evolution rate, reaching nearly 6000 µmol g^−1^ h^−1^ at 500 nm. This outstanding activity can be rationalized by the formation of an efficient heterojunction among TiO_2_, MoS_2_, and rGO, which synergistically enhances charge separation, interfacial charge transfer, and visible-light absorption. TiO_2_ serves as a stable wide-bandgap photocatalyst with strong UV absorption, while rGO provides a conductive platform that facilitates electron mobility, reduces charge recombination, and promotes light harvesting through its extended π-conjugated system [69]. The incorporation of MoS_2_ introduces a narrow-bandgap semiconductor with well-known catalytic activity for the hydrogen evolution reaction (HER), which then acts as a co-catalyst, offering abundant active edge sites and lowering the overpotential required for proton reduction [70]. The observed activity trend—5% MoS_2_ > 3% MoS_2_ > 10% MoS_2_ > 1% MoS_2_ > P25-rGO—clearly indicates that a moderate MoS_2_ loading is optimal for balancing these effects. At 1% MoS_2_, the number of catalytically active sites is likely insufficient to significantly improve HER kinetics, whereas an excessive amount of MoS_2_ (e.g., 10%) could lead to detrimental effects such as nanoparticle agglomeration, light shielding, and partial coverage of TiO_2_ or rGO surfaces, thereby impeding photon absorption and reducing charge accessibility. Additionally, the decline in activity observed beyond 500 nm is consistent with the intrinsic bandgap limitations of the semiconductor components, as the photon energy becomes inadequate to excite electrons from the valence to the conduction band. These findings support that the careful modulation of MoS_2_ content within the ternary composite is essential to maximize photocatalytic efficiency, and they emphasize the importance of interfacial engineering, band alignment, and light absorption optimization in designing next-generation nanostructured materials for sustainable hydrogen production.

**Figure 12:**
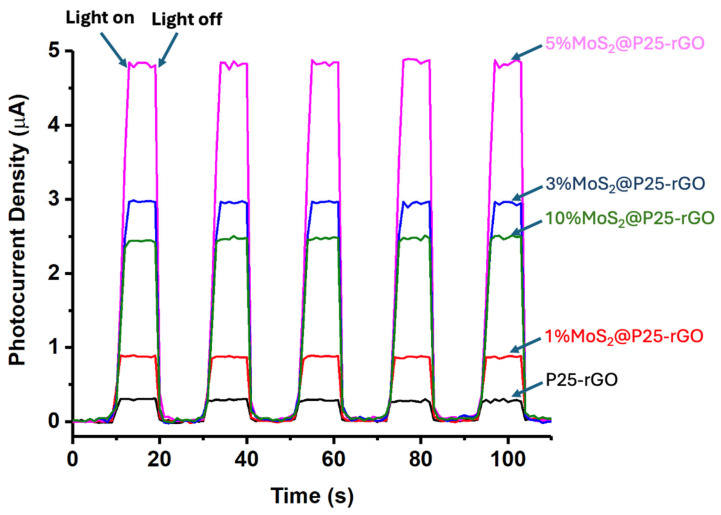
Transient photocurrent response in the light-on−light-off processes of the synthesized catalysts under irradiation at 500 nm.

The long-term performance and mechanistic aspects of the photocatalytic hydrogen evolution reaction (HER) using MoS_2_@P25-rGO composites were further examined through a series of complementary experiments, including transient photocurrent measurements, scavenger assays, and recyclability tests. The photocurrent responses under chopped light illumination (Figure 12) provide direct insight into the efficiency of photogenerated charge separation and transport. The 5% MoS_2_@P25-rGO catalyst exhibited the highest photocurrent density (~5 μA), followed by 3% MoS_2_@P25-rGO, 10% MoS_2_@P25-rGO, 1% MoS_2_@P25-rGO, and finally the unmodified P25-rGO. This order of photocurrent response precisely mirrors the trend observed in photocatalytic hydrogen production (Figure 11) and also aligns with the photocatalytic activity for malathion degradation discussed in earlier sections. These consistent trends across multiple techniques confirm that the enhanced photoactivity of the 5% MoS_2_ composite is directly linked to its superior charge separation and transport characteristics, which are facilitated by the synergistic interactions among MoS_2_, P25, and rGO.

To further clarify the mechanistic pathway of HER, radical scavenger experiments were conducted using EDTA-Na_2_, a known hole scavenger (see Figure S5) [44]. The addition of EDTA resulted in a significant enhancement in H_2_ production across the tested wavelengths compared to the control without the scavenger. This suggests that photogenerated holes act as recombination centers or engage in parallel oxidative reactions, and that their suppression enables a higher fraction of electrons to participate in proton reduction. These results support the hypothesis that MoS_2_ not only facilitates electron transfer but also serves as an efficient co-catalyst for proton reduction, with rGO acting as an electron mediator that enhances interfacial conductivity [15,71].

In terms of practical application, the recyclability of the 5% MoS_2_@P25-rGO photocatalyst was evaluated over 10 consecutive HER cycles (Figure S6). The system retained 91.9% of its initial hydrogen production capacity after 10 uses, with a performance drop of only 8.1%. This photostability underscores the structural robustness of the heterostructure and the durability of the active sites, confirming the feasibility of this material for long-term solar hydrogen generation. The strong interfacial bonding among MoS_2_, TiO_2_, and rGO components likely prevents leaching or deactivation, maintaining catalytic integrity over multiple uses. Taken together, these findings demonstrate the strong correlation among photocatalytic performance, charge transport efficiency, and material stability, positioning 5% MoS_2_@P25-rGO as a promising candidate for sustainable hydrogen production.

**Figure 13:**
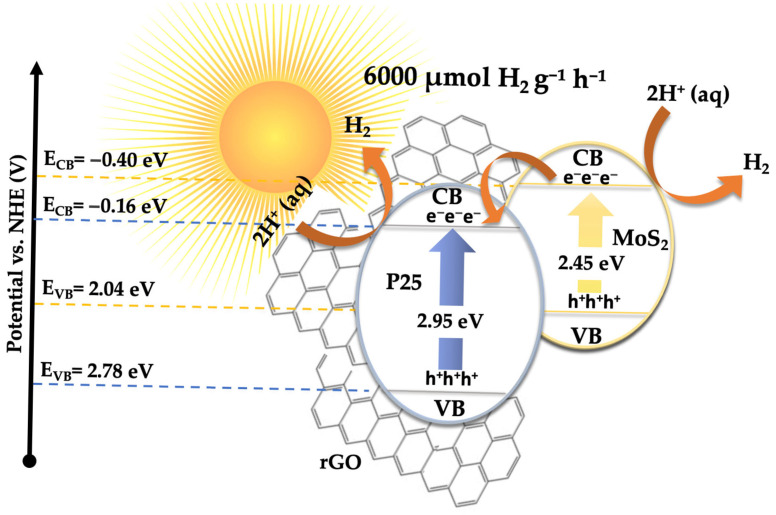
Schematic diagram of the proposed mechanism for hydrogen production under irradiation.

Based on the previously discussed results, a plausible mechanism has been proposed to explain the photocatalytic hydrogen evolution activity, consistent with the experimental observations (see Figure 13). The outstanding H_2_ production performance of the 5% MoS_2_@P25-rGO photocatalyst arises from the interplay among its three constituents—TiO_2_ (P25), MoS_2_, and reduced graphene oxide (rGO)—which together form a hierarchical heterostructure capable of efficient light absorption, charge separation, and catalytic functionality under visible-light irradiation. Among them, MoS_2_ acts as the primary absorber of visible light. Upon irradiation, electrons are promoted from its valence band (VB) to its conduction band (CB), leaving behind photogenerated holes. TiO_2_ (P25), with a wider bandgap (~3.2 eV), is less responsive to visible light; however, the incorporation of MoS_2_ and rGO into the structure redshifts the optical absorption of the composite, allowing some activation of TiO_2_ under solar-simulated conditions. Moreover, interfacial interactions can induce localized mid-gap states, enhancing visible-light response. Band edge calculations based on Mulliken’s electronegativity theory suggest that the CB potential of MoS_2_ (–0.405 eV vs. NHE) is more negative than that of TiO_2_ (–0.165 eV), while TiO_2_ has a more positive VB (+2.785 eV), making it a potent oxidant. This band alignment favors a directional flow of charge carriers: electrons generated in TiO_2_ or MoS_2_ transfer toward MoS_2_ and rGO, while holes accumulate on TiO_2_ [72]. Additionally, rGO acts as a conductive electron mediator that bridges MoS_2_ and TiO_2_, facilitating ultrafast charge transfer and delocalization, while also serving as a high-surface-area scaffold for active site dispersion [39]. This spatial charge separation is further evidenced by the strong quenching of photoluminescence (PL) in the composite and its enhanced transient photocurrent response, which indicate suppressed electron–hole recombination. The 5% MoS_2_@P25-rGO composite shows the highest photocurrent density and the lowest PL intensity among all tested samples, consistent with its superior H_2_ production rates. At the MoS_2_ surface, electrons reduce protons (H^+^) from the solution to generate H_2_, taking advantage of the abundant and catalytically active edge sites on MoS_2_. Meanwhile, the holes in TiO_2_ oxidize sacrificial agents added to the solution, preventing recombination and sustaining the redox cycle. Scavenger experiments confirm that hole consumption significantly enhances H_2_ evolution, highlighting the importance of maintaining separate pathways for electrons and holes. This mechanism is consistent with that proposed for malathion degradation, where the same spatial charge separation and vectorial charge migration were identified. In the absence of oxygen, electrons that would otherwise reduce O_2_ (to form ·O_2_^−^ for oxidative degradation) are now fully available for proton reduction, thus explaining the high H_2_ evolution rates. The rGO sheets not only improve the conductivity and dispersibility of MoS_2_ but also ensure intimate contact among the components, which is essential for maintaining an efficient interfacial electric field and continuous charge flow [73,74]. Overall, the 5% MoS_2_@P25-rGO catalyst operates via a cooperative mechanism that combines light absorption, charge generation, and catalytic functionality across its components. The result is a system capable of exploiting a broad portion of the solar spectrum while maintaining low recombination losses and high redox activity, delivering significant hydrogen generation rates and demonstrating its promise for sustainable solar fuel applications.

## 4. Conclusions

In this study, a series of MoS_2_-decorated TiO_2_-rGO ternary nanocomposites were successfully synthesized and extensively characterized to evaluate their dual functionality in photocatalytic malathion degradation and hydrogen evolution under UV-visible irradiation. The hybrid photocatalysts were prepared by integrating exfoliated MoS_2_ nanosheets with TiO_2_ nanoparticles supported on reduced graphene oxide, creating intimate heterojunctions designed to enhance light absorption, charge separation, and surface reactivity. Among the catalysts tested, the composite containing 5 wt% MoS_2_ exhibited the highest photocatalytic performance in both applications, confirming the critical importance of compositional balance and interfacial engineering in these systems. Comprehensive structural, morphological, and electronic analyses revealed that all nanocomposites maintained the anatase phase of TiO_2_ while incorporating well-dispersed rGO and MoS_2_. Raman spectroscopy and XRD confirmed the phase purity and successful integration of all components without the formation of undesirable secondary phases. HRTEM and SEM analyses illustrated the morphological coherence and homogeneous dispersion of the layered materials, while BET analysis demonstrated a marked increase in surface area with the inclusion of rGO and MoS_2_. Interestingly, despite having the highest surface area, the 10% MoS_2_-loaded composite underperformed in both degradation and hydrogen evolution, indicating that excessive MoS_2_ can hinder photocatalytic efficiency by introducing recombination centers or limiting photon penetration due to light shielding or overcoating of active sites. Optical studies provided further insights into the behavior of the ternary systems. UV-Vis DRS demonstrated a systematic redshift and bandgap narrowing with increasing MoS_2_ content, enhancing visible-light absorption. PL measurements revealed a significant suppression of electron–hole recombination, particularly in the 5% MoS_2_@TiO_2_-rGO sample, suggesting a highly efficient charge separation mechanism facilitated by the synergistic roles of rGO as an electron mediator and MoS_2_ as an electron acceptor and catalytic site. XPS confirmed the stability of the Ti^4^^+^ and Mo^4^^+^ oxidation states, as well as interfacial electronic interactions indicative of covalent or electrostatic coupling among the components. Photocatalytic degradation of malathion revealed clear performance trends across the different composites, with the 5% MoS_2_ catalyst achieving nearly complete degradation within 60–90 min. GC-MS analysis identified multiple intermediate products and proposed five principal degradation pathways involving hydrolysis, desulfuration, demethylation, and oxidative ring-opening, supported by radical scavenger experiments, which established superoxide radicals (·O_2_^−^) as the main active species. The optimal photocatalytic performance was linked to the ability of the heterostructure to promote efficient interfacial charge separation and favor a Z-scheme or Type-II electron transfer mechanism. Additionally, recyclability tests confirmed that the best-performing catalyst retained more than 95% of its activity over 10 consecutive cycles, underscoring its structural stability and long-term applicability in continuous operation scenarios. In the hydrogen evolution reaction (HER), similar trends were observed. The photocatalytic H_2_ production peaked at 5% MoS_2_ loading, achieving yields of nearly 6000 µmol·g^−1^·h^−1^ under neutral pH conditions and optimal catalyst dosage (1.0 g·L^−1^). Photocurrent measurements, radical trapping studies, and recyclability tests supported the same conclusions drawn for malathion degradation: that effective charge separation and transport are crucial for photocatalytic efficiency, and that the ternary synergy among TiO_2_, MoS_2_, and rGO plays a pivotal role. Notably, the catalyst’s photocurrent generation profile mirrored both HER and pollutant degradation efficiencies, providing strong evidence of consistent structure–function relationships. Importantly, this study extends beyond fundamental materials development by demonstrating clear pathways to practical implementation. The photocatalysts developed not only exhibit high performance in model reactions but also operate under conditions relevant to environmental remediation and sustainable fuel production, including neutral pH, ambient temperature, and moderate light intensity. These factors are critical for real-world applicability, as they ensure compatibility with existing water treatment and solar-driven hydrogen generation infrastructure.

Altogether, this work demonstrates that careful modulation of MoS_2_ content in TiO_2_-rGO nanocomposites results in significantly improved photocatalytic activity for both pollutant removal and renewable energy production. The 5% MoS_2_@TiO_2_-rGO nanocomposite represents an optimal configuration for exploiting visible-light-induced processes via enhanced interfacial charge transfer, light harvesting, and active site accessibility. These findings not only highlight the versatility of MoS_2_-based ternary photocatalysts but also emphasize their direct relevance to scalable technologies for water decontamination and solar hydrogen production. By bridging mechanistic insights with real-world performance metrics, the present work provides a robust framework for designing next-generation multifunctional photocatalysts tailored to pressing environmental and energy challenges.

## Figures and Tables

**Table 1:** Band structure parameters used for mechanism estimation. rGO is not included in the table, as it acts primarily as a conductive support without discrete band edges.

Material	Absolute Electronegativity χ (eV)	Optical Bandgap Eg (eV)	E_CB_ (eV)	E_VB_ (eV)
P25-rGO	5.81	2.95	–0.165	+2.785
MoS_2_	4.995	1.80	–0.405	+2.045

## Data Availability

The original contributions presented in this study are included in the article/Supplementary Material. Further inquiries can be directed to the corresponding author(s).

## Supplementary Materials

The following supporting information can be downloaded at https://www.mdpi.com/article/10.3390/ma18112602/s1. Figure S1: Evaluation of the initial concentration of 5% MoS_2_@P25-rGO on the photodegradation of malathion (**a**), effect of pH on the photocatalytic activity of the 5% MoS_2_@P25-rGO catalyst for photodegradation of malathion (**b**), and control experiments for 5% MoS_2_@P25-rGO with malathion under irradiation (**c**). All experiments were performed in triplicate. Error bars in (**a**) and (**b**) represent standard deviations, with observed variability below 4%. In (**c**), error bars were omitted to preserve visual clarity due to overlapping data from multiple catalysts. Figure S2: Photodegradation of malathion by 5% MoS_2_@P25-rGO in the presence of different scavengers. All experiments were performed in triplicate. Although data variability was consistently below 4%, error bars were omitted to preserve visual clarity due to the overlap of data series. Figure S3: Recyclability of 5% MoS_2_@P25-rGO after 10 consecutive catalytic cycles of photodegradation of malathion under irradiation. All experiments were performed in triplicate. Error bars represent standard deviations, with observed variability below 4%. Figure S4: Evaluation of the initial concentration of 5% MoS_2_@P25-rGO on the efficiency of hydrogen production (**a**), effect of pH on the photocatalytic activity of the 5% MoS_2_@P25-rGO catalyst for hydrogen production (**b**), and control experiments for 5% MoS_2_@P25-rGO on the efficiency of hydrogen production (**c**). Each experiment was independently repeated three times. The error bars indicate standard deviations, with data variability remaining below 5% in all cases. Figure S5: Hydrogen production via water splitting using 5% MoS_2_@P25-rGO under irradiation and also in the presence of EDTA-Na_2_. All measurements were conducted in triplicate under identical conditions. The error bars reflect the standard deviation, with variations not exceeding 5% throughout. Figure S6: Recyclability of 5% MoS_2_@P25-rGO after 10 consecutive catalytic cycles of hydrogen production under irradiation at 500 nm. All experiments were performed in triplicate. Error bars represent standard deviations, with observed variability below 5%. Table S1: BET surface area of the synthesized materials. Table S2: apparent quantum efficiency (AQE) calculation details. The following table summarizes the experimental conditions, physical constants, and step-by-step calculations used to determine the AQE value for the 5% MoS_2_@P25-rGO composite under monochromatic irradiation at 500 nm.
