# Supplementary material for: From Pollutant Removal to Renewable Energy: MoS2-Enhanced P25-Graphene Photocatalysts for Malathion Degradation and H2 Evolution
Source: Materials (Basel). 2025 Jun 3;18(11):2602. doi: 10.3390/ma18112602 (PMC12156005; doi:10.3390/ma18112602)
Supplement: Supplementary file 1 [file materials-18-02602-s001.zip › materials-3644802-supplementary.pdf]

Supporting Information

From Pollutant Removal to Renewable Energy: MoS_2-Enhanced P25-Graphene Photocatalysts for Malathion Degradation and H_2 Evolution

Cristian Martínez-Perales [1], Abniel Machín [2,*], Pedro J. Berríos-Rolón [1], Paola Sampayo [1], Enrique Nieves [3], Loraine Soto-Vázquez [4], Edgard Resto [4], Carmen Morant [5], José Ducongé [1], María Cotto [1], and Francisco Márquez [1,*]

[1]Nanomaterials Research Group, Department of Natural Sciences and Technology, Division of Natural Sciences, Technology and Environment, Universidad Ana G. Méndez-Gurabo Campus, 00778PR, USA
[2]Environmental Catalysis Research Lab, Division of Science, Technology and Environment, Cupey Campus, Universidad Ana G. Méndez, Cupey, PR 00926, USA
[3]Department of Pharmaceutical Sciences, Nova Southeastern University, Puerto Rico Campus, San Juan, 00926PR, USA
[4]Materials Characterization Center Inc., Molecular Sciences Research Center, University of Puerto Rico, San Juan, PR 00926, USA
[5]Department of Applied Physics, Autonomous University of Madrid, and Instituto de Ciencia de Materiales Nicolás Cabrera, 28049, Madrid, Spain

*Correspondence: machina1@uagm.edu (A.M.); fmarquez@uagm.edu (F.M.); Tel.: +1-787-878-2612 (ext. 220) (A.M.); +1-787-743-7979, ext. 4250 (F.M.)

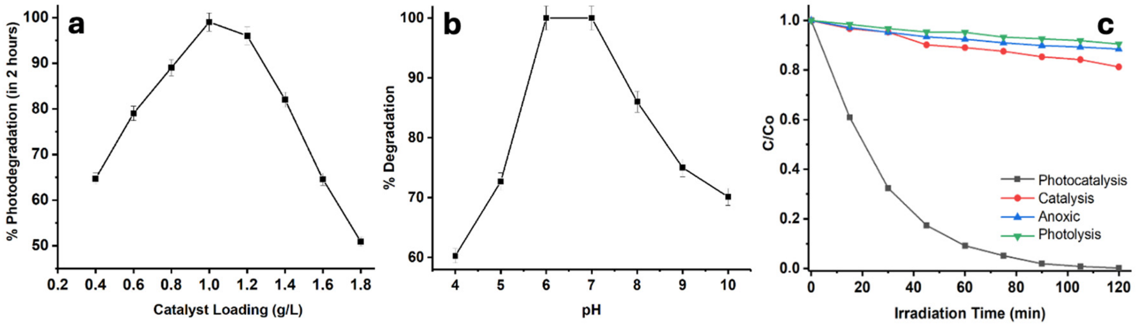

Figure S1. Evaluation of the initial concentration of 5%MoS_2@P25-rGO on the photodegradation of malathion (a); effect of pH on the photocatalytic activity of the 5%MoS_2@P25-rGO catalyst for photodegradation of malathion (b); and control experiments for 5%MoS_2@P25-rGO with malathion under irradiation (c). All experiments were performed in triplicate. Error bars in (a) and (b) represent standard deviations, with observed variability below 4%. In (c), error bars were omitted to preserve visual clarity due to overlapping data from multiple catalysts.

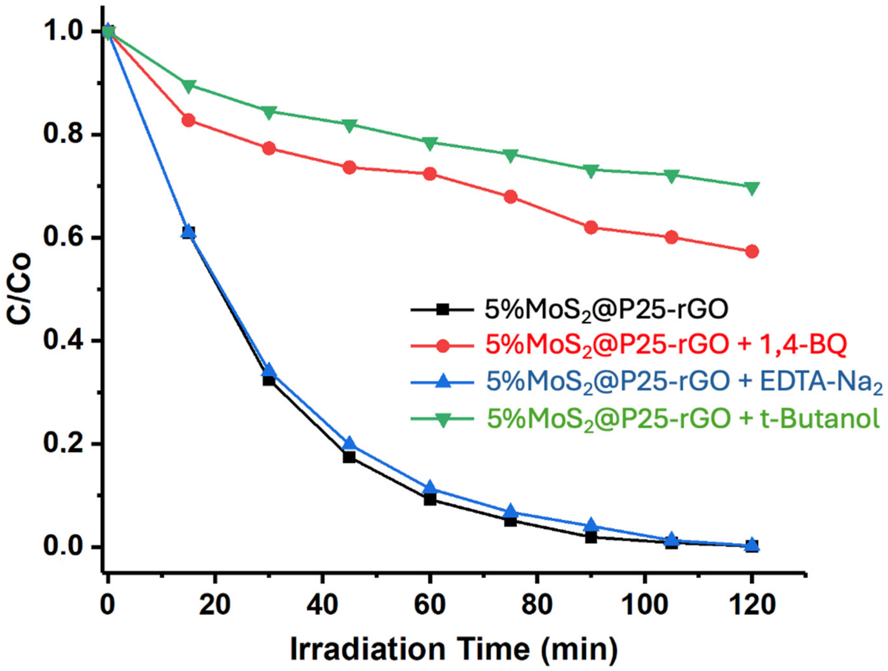

Figure S2. Photodegradation of malathion by 5%MoS_2@P25-rGO in the presence of different scavengers. All experiments were performed in triplicate. Although data variability was consistently below 4%, error bars were omitted to preserve visual clarity due to the overlap of data series.

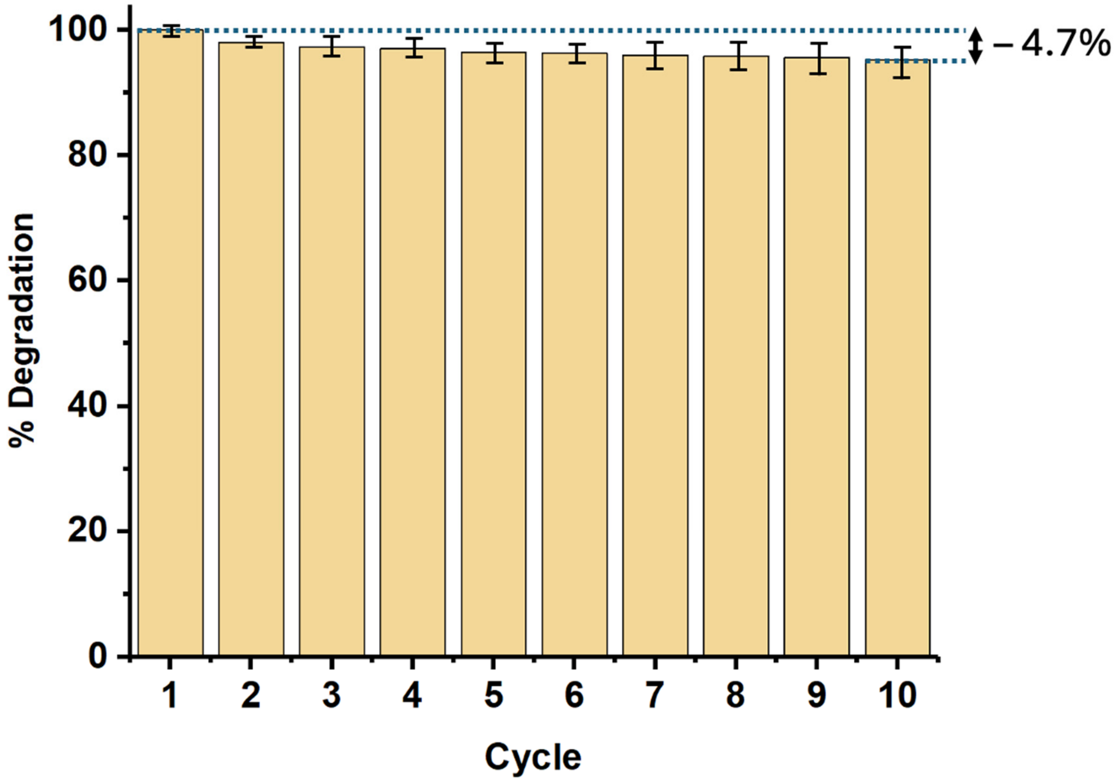

Figure S3. Recyclability of 5%MoS_2@P25-rGO after 10 consecutive catalytic cycles of photodegradation of malathion under irradiation. All experiments were performed in triplicate. Error bars represent standard deviations, with observed variability below 4%.

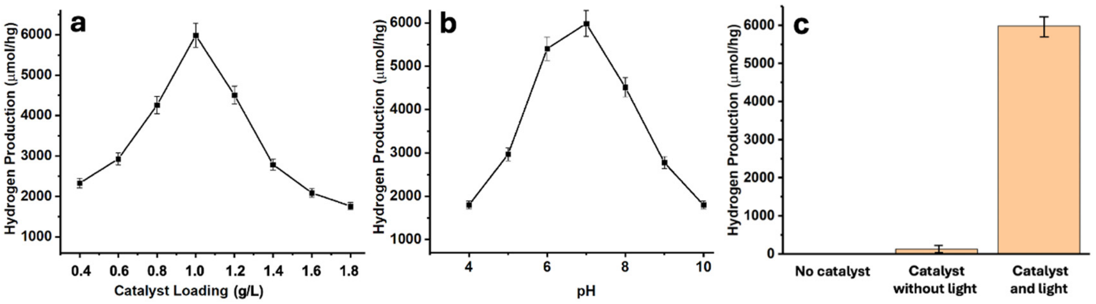

Figure S4. Evaluation of the initial concentration of 5%MoS_2@P25-rGO on the efficiency of hydrogen production (a); effect of pH on the photocatalytic activity of the 5%MoS_2@P25-rGO catalyst for hydrogen production (b); and control experiments for 5%MoS_2@P25-rGO on the efficiency of hydrogen production (c). Each experiment was independently repeated three times. The error bars indicate standard deviations, with data variability remaining below 5% in all cases.

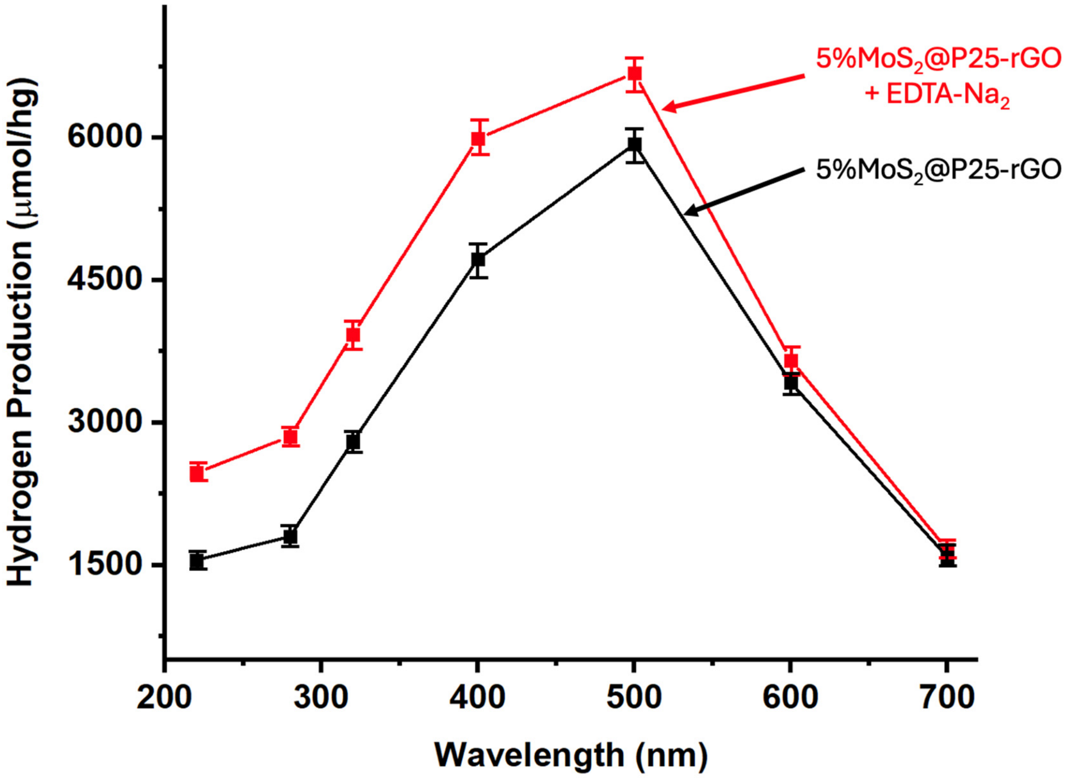

Figure S5. Hydrogen production via water splitting using 5%MoS_2@P25-rGO under irradiation and in the presence of EDTA-Na_2. All measurements were conducted in triplicate under identical conditions. The error bars reflect the standard deviation, with variations not exceeding 5% throughout.

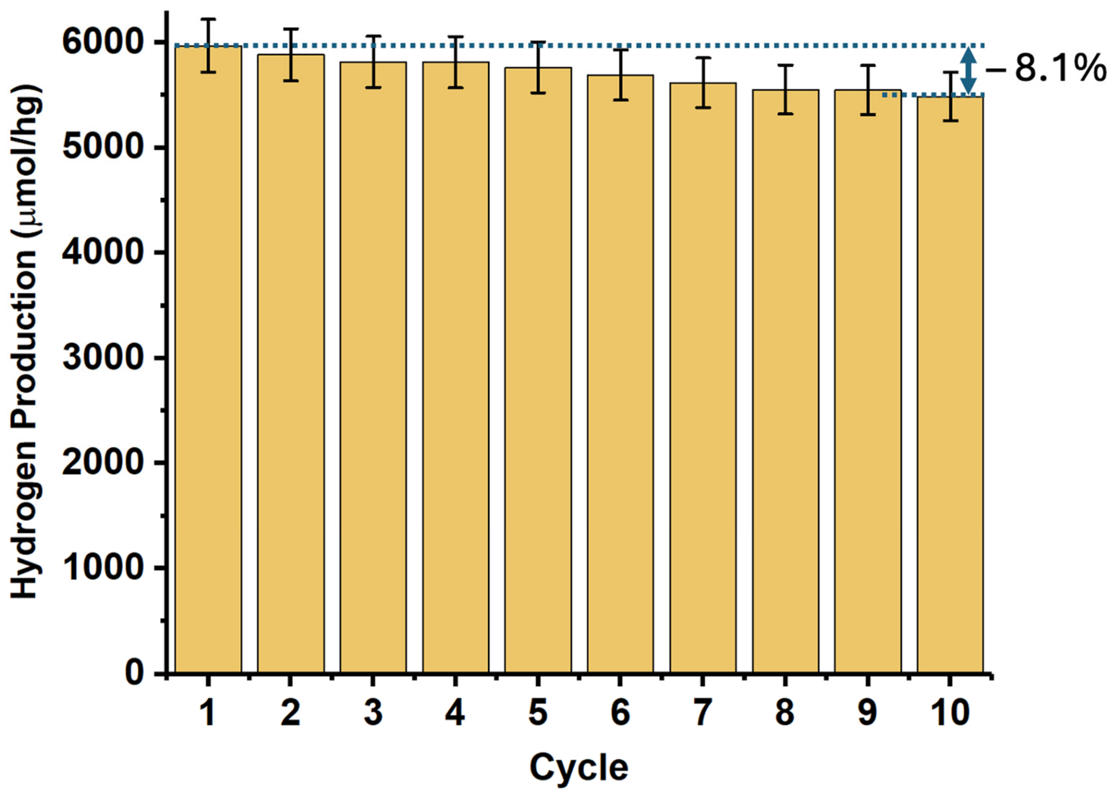

Figure S6. Recyclability of 5%MoS_2@P25-rGO after 10 consecutive catalytic cycles of hydrogen production under irradiation at 500 nm. All experiments were performed in triplicate. Error bars represent standard deviations, with observed variability below 5%.

Table S1. BET surface area of the synthesized materials.

Material	BET area (m^2/g)
P25	48
rGO	429
P25-rGO	483
1%MoS_2@P25-rGO	492
3%MoS_2@P25-rGO	496
5%MoS_2@P25-rGO	503
10%MoS_2@P25-rGO	521

Table S2. Apparent quantum efficiency (AQE) calculation details. The following table summarizes the experimental conditions, physical constants, and step-by-step calculations used to determine the AQE value for the 5%MoS_2@P25-rGO composite under monochromatic irradiation at 500 nm.

Parameter / Step	Value / Description
Catalyst mass	50 mg = 0.050 g
Irradiated area	10 cm^2
Irradiation time	2 hours = 7200 s
Light intensity at 500 nm	120 $mW\cdot cm^{-2}$
Hydrogen evolution rate	6000 $\mu mol\cdot g^{-1}\cdot h^{-1}$
Total H_2 evolved	600 μmol = 6.00×10^{-4} mol
Avogadro's number	6.022×10^{23} mol^{-1}
H_2 molecules evolved	3.61×10^{20} molecules
Photon energy at 500 nm	3.976×10^{-19} J
Total power	1.2 W (0.12 W/cm^2 × 10 cm^2)
Total energy incident	8640 J (1.2 W × 7200 s)
Incident photons	2.17×10^{22} photons
AQE (%)	3.33%
